# Excited State Branching Processes in a Ru(II)‐Based Donor–Acceptor–Donor System

**DOI:** 10.1002/chem.202404671

**Published:** 2025-05-03

**Authors:** Guangjun Yang, Louis Blechschmidt, Linda Zedler, Clara Zens, Kamil Witas, Maximilian Schmidt, Birgit Esser, Sven Rau, Georgina E. Shillito, Benjamin Dietzek‐Ivanšić, Stephan Kupfer

**Affiliations:** ^1^ Institute of Physical Chemistry Friedrich Schiller University Jena Helmholtzweg 4 07743 Jena Germany; ^2^ Department Spectroscopy and Imaging ‐ Work group Photophysics and Photochemistry of Functional Interfaces Leibniz Institute of Photonic Technology (IPHT) Albert‐Einstein‐Straße 9 07745 Jena Germany; ^3^ Institute for Inorganic Chemistry 1 Ulm University 89081 Ulm Germany; ^4^ Institute of Organic Chemistry II and Advanced Materials Ulm University Albert‐Einstein‐Allee 11 89081 Ulm Germany; ^5^ Present address: Leibniz Institute of Surface Engineering e.V. (IOM) Permoserstrasse 15 04318 Leipzig Germany

**Keywords:** dissipative quantum dynamics, femtosecond transient spectroscopy, interference effects, photoinduced electron transfer, semiclassical Marcus theory

## Abstract

Excited state properties such as excitation energy, accessibility of the respective excited state either by direct or indirect population transfer, and its lifetime govern the application of these excited states in light‐driven reactions, for example, photocatalysis using transition metal complexes. Compared with triplet metal‐to‐ligand charge transfer (^3^MLCT) states, charge‐separated (^3^CS) excited states involving organic moieties, such as triplet intra‐ligand or ligand‐to‐ligand charge transfer (^3^ILCT and ^3^LLCT) states, tend to possess longer‐lived excited states due to the weak spin‐orbit coupling with the closed‐shell ground state. Thus, the combination of inorganic and organic chromophores enables isolating the triplet states onto the organic chromophore. In this study, we aim to elucidate the excited‐state relaxation processes in a Ru(II)‐terpyridyl donor–acceptor–donor system (**RuCl**) in a joint spectroscopic‐theoretical approach combining steady‐state and time‐resolved spectroscopy as well as quantum chemical simulations and dissipative quantum dynamics. The electron transfer (ET) processes involving the low‐lying ^3^MLCT, ^3^ILCT, and ^3^LLCT excited states were investigated experimentally and computationally within a semiclassical Marcus picture. Finally, dissipative quantum dynamical simulations—capable of describing incomplete ET processes involving all three states—enabled us to unravel the competitive relaxation channels at short and long timescales among the strongly coupled ^3^MLCT‐^3^ILCT states and weakly coupled ^3^MLCT‐^3^LLCT and ^3^ILCT‐^3^LLCT states.

## Graphical abstract

The photoinduced electron transfer processes in a Ru(II)‐based push–pull–push architecture which combines an inorganic and organic chromophore are elucidated in a joint spectroscopic‐theoretical approach. The applied design concept allows the excited state to be localized away from the metal onto the organic chromophore.

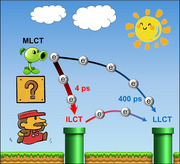

## 1 Introduction

Solar energy conversion is one of the most promising strategies for transitioning toward sustainable energy.^[^ ^1^ ^]^ Supramolecular photocatalysis in particular, enables the transformation of sunlight into chemical energy, for example, into green hydrogen or converting carbon dioxide into hydrocarbon fuels such as formaldehyde, methanol, and formic acid.^[^ ^2^ ^]^ In these processes, the properties of the involved excited states play a crucial role in determining the photocatalytic behavior and potential applications, including their excitation energy, accessibility, and lifetime. In recent decades, 4d and 5d transition metal complexes, particularly Ru‐based chromophores, have been extensively investigated and developed due to their broad absorption features, comparably long‐lived charge‐separated (CS) states, high photostability, and tunable redox properties.^[^ ^3^ ^]^ In order to utilize such transition metal complexes in photochemical reactions, the CS excited state must be sufficiently long‐lived to enable efficient interaction with the reactant molecule(s). One compelling approach to avoid a rapid deactivation of the CS species, that is, from a triplet metal‐to‐ligand charge transfer (^3^MLCT) state back to the singlet ground state (GS), is to integrate organic and inorganic chromophores into a single structure. This strategy enables panchromatic absorption in the visible region based on both the inorganic as well as the organic chromophore. Furthermore, this concept allows us to overcome the design tension between the creation of long‐lived excited (triplet) states and the population of such states. In other words, typically, long‐lived triplet states have low spin‐orbit coupling (SOC), and hence their population, either by direct photoexcitation or via subsequent excited‐state relaxation is low. Therefore, a long‐lived triplet state can be populated, but inefficiently. If the SOC is increased, the triplet state population efficiency is improved, which comes at the cost of a reduced excited‐state lifetime.^[^ ^4^ ^]^ Here, the combination of inorganic and organic chromophores into a push–pull–push architecture allows efficient intersystem crossing (ISC) to the triplet manifold via the inorganic chromophore (^3^MLCT state), followed by shifting the ^3^CS state away from the metal onto the organic chromophore, that is, forming long‐lived ^3^ππ* states, which are only weakly coupled to the ^1^GS.^[^ ^5^ ^]^

**Figure 1:**
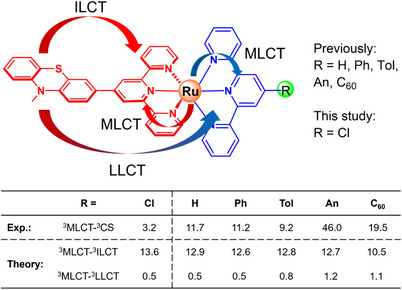
Structures of previously^[^ ^5d,e^ ^]^ (in dichloromethane) and herein studied Ru(II)‐terpyridyl complexes (RuCl, R = Cl; in acetonitrile) incorporating a phenothiazine‐tpy donor ligand (red) as well as a tpy‐based acceptor ligand (blue). Prominent electronic transitions are indicated. Experimentally, temperature‐dependent pump‐probe spectroscopy determined a remote control effect of the electronic coupling (in meV) between the lowest ^3^MLCT and an unidentified triplet charge separated (^3^CS) state by structural variation of R.^[^ ^5d^ ^]^ Quantum chemical simulations focused on identifying the nature of this ^3^CS state and revealed strongly coupled yet structural independent ^3^ILCT acceptor states and weakly coupled yet structural dependent ^3^LLCT acceptor states along the series of Ru(II) complexes.^[^ ^5e^ ^]^

Technically, the population transfer efficiency from such a ^3^MLCT state to the desired ^3^CS state can be tailored by the introduction of electron‐donating or electron‐withdrawing groups, which allows modulation of the thermodynamical properties, that is, the driving force (Δ*G*) and reorganization energy (*λ*) of the process. Notably, this strategy also allows tuning of the excited‐state electron‐transfer (ET) processes in favor of populating the target ^3^CS state. However, the introduced substitution may lead to a scenario where the energy of the long‐lived state is insufficient to drive the subsequent desired photochemical reaction.^[^ ^6^ ^]^ An alternative and more sophisticated strategy is to modify the electronic coupling (*V*_DA_) between the involved electronic excited states (e.g., ^3^MLCT and ^3^CS). Thereby, the kinetics of the excited‐state processes are tuned in favor of the desired charge‐separated species without “losing” energy. In this context, joint spectroscopic‐theoretical as well as computational investigations, for example, by the groups Weinstein^[^ ^7^ ^]^ and Daniel et al.,^[^ ^8^ ^]^ focused on a class of square–planar Pt(II) complexes, which incorporate in trans‐arrangement an organic phenothiazine (PTZ) donor as well as a naphthalene‐imide‐based (NDI) acceptor unit. Thereby, the electronic communication between the lowest energy ^3^MLCT and ^3^CS states— a triplet ligand‐to‐ligand charge transfer (^3^LLCT) state— in these PTZ‐Pt(II)‐NDI systems was found the be highly dependent on the relative orientation of the donor and acceptor units. This vibronic effect on the electronic coupling in these complexes is related to the rather flexible coordination environment in the vicinity of the Pt(II) center, in particular the rather free rotation of the donor and acceptor groups. In a related study, Luo et al.^[^ ^5e^ ^]^ investigated a family of Ru(II)‐terpyridyl donor–acceptor–donor systems, which incorporate a PTZ donor moiety on a terpyridyl (tpy) ligand and a series of substituents in the periphery of the second tpy ligand (see Figure 1) by means of conducting ns‐transient absorption spectroscopy and electrochemistry experiments.

Notably, the octahedral coordination sphere of the Ru(II) center leads to a ridged environment and to an almost perpendicular orientation of the PTZ‐tpy and tpy‐R ligands, while an additional charge transfer state is introduced, namely an intra‐ligand charge transfer (ILCT) state localized on the PTZ‐tpy ligand. The results obtained by Luo et al. demonstrated that the photoinduced ET kinetics are predominantly governed by the electronic coupling, with a lesser contribution from the underlying thermodynamic properties.^[^ ^5d^ ^]^ However, the precise nature of the ^3^CS, that is, ^3^LLCT, ^3^ILCT or even a combination of both states, remained unknown as the applied pump‐probe spectroscopy cannot differentiate between these two states, which merely differ in the excited electron either within the lowest $\pi_{\mathrm{tpy}}^{*}$ orbital of the PTZ‐tpy ligand or within the respective antibonding orbital of the tpy‐R ligand. In our recent theoretical investigation,^[^ ^5e^ ^]^ we focused on elucidating the kinetics of the ET‐branching channels in‐depth between the lowest‐energy ^3^MLCT state and the two ^3^CS states in these Ru(II) photosensitizers. Our quantum chemical simulations revealed the impact of the two different low‐lying ^3^CS states involving the ligand sphere of the Ru complexes, namely the ^3^ILCT and the ^3^LLCT state as described above. Thereby, the ^3^ILCT involves an ET from the PTZ group to the low‐lying π* orbitals of the linked tpy ligand, while the LLCT process is associated with a single‐electron transfer from the PTZ to the second tpy ligand. Our simulations revealed strong electronic couplings (*V*_DA_ ≈ 1.3 × 10^−2^ eV) and thus efficient ET from the ^3^MLCT to the ^3^ILCT states, yet the coupling between these states was found to be independent of the introduced substitution pattern in the periphery of the other tpy ligand. In contrast, the electronic communication between the ^3^MLCT and the ^3^LLCT states was revealed to be small as the involved molecular orbitals are almost orthogonal. Yet, the *V*_DA_ values (ranging from 4.5 × 10^−4^ to 11.5 × 10^−4^ eV) are highly sensitive to the substitution pattern. Thus, we speculate that the remote‐control effect of the electronic coupling, as inferred by Luo et al.^[^ ^5d^ ^]^ using temperature‐dependent pump‐probe spectroscopy, originates from an interference between the ^3^MLCT state and the two ^3^CS states of the organic chromophore (^3^ILCT and ^3^LLCT), which are indistinguishable based on the applied spectroscopic techniques. This way, the magnitude of the electronic coupling could be provided by the strongly coupled ^3^MLCT‐^3^ILCT states while its structure‐dependent modification of the R group (see Figure 1) is provided by the weakly coupled ^3^LLCT state. Therefore, elucidating the interaction and the partial population transfer among these three charge‐separated states is of potential importance to steer electron transfer processes, for example, in photocatalytic applications.

In the scope of the present work, we aim to elucidate the light‐driven processes and excited‐state relaxation cascades associated with the population of ^3^CS states (^3^ILCT and ^3^LLCT) in a **PTZ‐tpy‐Ru‐tpy‐Cl** (**RuCl**) push–pull–push structure (tpy = 2, 2′:6′, 2′′‐terpyridine; PTZ = 10‐methylphenothiazinyl), as shown in Figure 1. Thereby, the photophysical properties of **RuCl** are unraveled by quantum chemical and quantum dynamical simulations as well as steady‐state and time‐resolved spectroscopy. **RuCl** acts as a representative for the family of polypyridyl‐based transition metal complexes that incorporate an additional organic chromophore into the ligand sphere architecture. Following studies of the Franck–Condon photophysics, the excited state relaxation processes involving the low‐lying triplet states (^3^MLCT, ^3^ILCT, and ^3^LLCT) are investigated computationally using semiclassical Marcus theory as well as by dissipative quantum dynamical (DQD) simulations along efficient excited state reaction coordinates. The obtained insights with respect to the thermodynamics and the kinetics of the competitive ET processes are studied in a joint spectroscopic‐theoretical fashion. This way, population transfer between the ^3^MLCT and ^3^CS states (^3^ILCT and ^3^LLCT) and their interference as governed by the electronic coupling are elucidated.

## 2 Results and Discussion

The following section addresses the light‐driven processes for the present **RuCl** photosensitizer as predicted by time‐dependent density functional theory (TDDFT) and in conjunction with steady‐state and time‐resolved spectroscopy. Initially, structural and electronic properties within the Franck–Condon point as well as the nature of the electronic transitions underlying the UV–vis absorption bands are carefully evaluated. Subsequently, the kinetics of the photoinduced ET processes, populating the (long‐lived) ^3^CS species, that is, ^3^ILCT and ^3^LLCT as well as their dynamic interactions, are unraveled based on semiclassical Marcus theory and dissipative quantum dynamics and in synergy with electrochemical measurements as well as fs transient absorption (TA) spectroscopy.

### 2.1 Franck–Condon Photophysics

**Figure 2:**
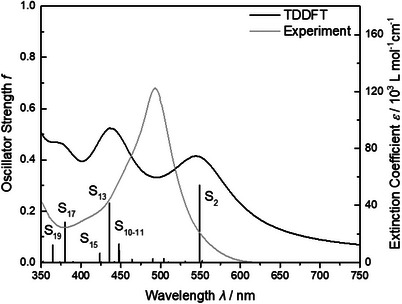
Experimental (grey) and simulated (black) UV–vis absorption spectrum of **RuCl** in CH_3_CN. TDDFT‐predicted transitions are broadened by Lorentzian functions with a full width at half maximum of 0.2 eV.

The Franck–Condon photophysics of the Ru(II) complex **RuCl**, were evaluated using UV–vis spectroscopy and TDDFT simulations. Figure 2 shows the experimental electronic absorption spectrum in CH_3_CN alongside the simulated spectrum.

According to our TDDFT simulations, the visible region of the electronic absorption spectrum of **RuCl** is dominated by a low‐lying and strongly dipole‐allowed ^1^ILCT transition at 2.26 eV (S_2_ at 549 nm, Table S6) as well as by a set of ^1^MLCT transitions with slightly higher excitation energies. Notably, the lowest energy excitation (S_1_; 2.25 eV, 552 nm) is characterized to be a dipole‐forbidden ^1^LLCT. Excitation into the ^1^MLCT states S_10_ (2.77 eV, 448 nm), S_11_ (2.77 eV, 448 nm), S_13_ (2.84 eV, 436 nm), and S_15_ (2.93 eV, 424 nm), respectively, leads to a population transfer from the t_2g_ orbitals (d_xy_, d_xz_, and d_yz_) into the low‐lying $\pi_{\mathrm{tpy}}^{*}$ orbitals of both terpyridyl ligands. In contrast, S_17_ (3.26 eV, 380 nm) and S_19_ (3.40 eV, 365 nm) feature mixed ^1^ILCT/^1^MLCT character, while the excited electron is primarily localized on the PTZ‐substituted tpy ligand. These results are consistent with our previous findings obtained for a series of structurally closely related Ru(II)‐based complexes (see Figure 1), as investigated by state‐of‐the‐art multiconfigurational calculations and TDDFT using hybrid, range‐separated and double‐hybrid functionals.^[^ ^5e^ ^]^ Consistently, all employed theoretical approaches predict a low‐lying ^1^ILCT and a higher lying ^1^MLCT bands, while the visual agreement with the experimental UV–vis spectrum, in particular in the 500 nm region seems improvable.

**Figure 3:**
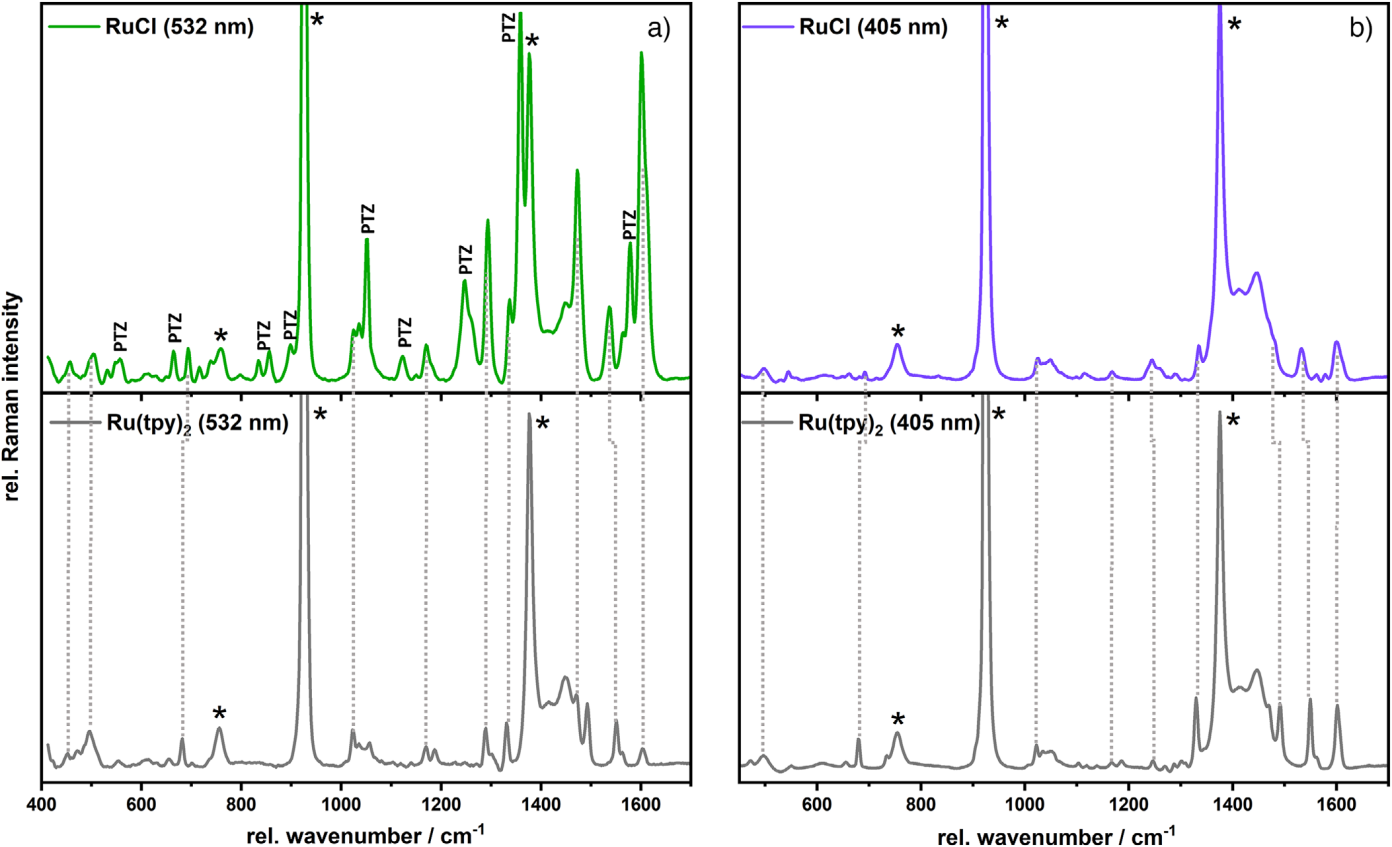
Wavelength‐dependent resonance Raman (rR) spectra of **RuCl** (CH_3_CN), excited at 532 nm (a, top) and 405 nm (b, top) in comparison with the rR spectra of **[Ru(tpy)_2_]^2+^** (bottom row), respectively. The applied Raman excitation wavelength at 405 nm matches the lowest energy ^1^MLCT absorption band of **RuCl**, whereas at 532 nm an ^1^ILCT from the PTZ unit to the adjacent tpy ligand is excited. Vibrational resonances of **[Ru(tpy)_2_]^2+^** are assigned to tpy modes in the rR spectrum of **RuCl** and marked with vertical dotted lines. PTZ associated rR vibrations are labelled, solvent bands are indicated by asterisks. All spectra are normalized to the solvent band (CH_3_CN) at 923 cm^−1^.

To corroborate the analysis of the Franck–Condon region, resonance Raman (rR) spectroscopy was employed.^[^ ^9^ ^]^ The rR spectra of **RuCl** were recorded in CH_3_CN with excitation wavelengths of 405 and 532 nm, that is, within the blue and the red flank of the absorption maximum of **RuCl** (Figure 3). Upon 532 nm excitation the rR spectrum is characterized by prominent bands at 549, 693, 834, 898, 1050, 1123, 1248, 1357, and 1579 cm^−1^ (Figure 3a, top). These bands can be assigned to PTZ‐based vibrations.^[^ ^10^ ^]^ In addition to PTZ‐associated bands, tpy vibrations at 1022, 1470, 1550, and 1602 cm^−1^ were also observed, identified by comparison with the rR spectrum of the homoleptic complex **[Ru(tpy)_2_]^2+^** (Figure 3a, bottom).^[^ ^11^ ^]^ The detection of both PTZ and tpy modes upon excitation of the ^1^ILCT transition is due to the fact that both, PTZ donor and tpy acceptor orbitals ($\pi_{\mathrm{PTZ}}$ and $\pi_{\mathrm{tpy}}^{*}$) contribute to the resonance enhancement of the vibrational modes in the rR spectrum. This experimental finding reflects the theoretical results and the impact of the energetically low‐lying and strongly dipole‐allowed ^1^ILCT transition (into S_2_ at 549 nm) from the electron‐rich PTZ group to the linked tpy moiety. In contrast, the rR spectrum of **RuCl** excited at 405 nm is dominated exclusively by tpy modes when compared with the rR pattern of **[Ru(tpy)_2_]^2+^** (Figure 3b). This clearly shows that the ^1^MLCT absorption at 405 nm consists of Ru‐to‐tpy transitions, in particular with excitations into the S_11_ and S_13_^1^MLCT states (at 448 and 436 nm), as predicted at the TDDFT level of theory. Therefore, the excitation wavelength‐dependent rR data agrees with the quantum chemical results. Notably, the simulated excitation energy of the ^1^ILCT state is slightly underestimated by roughly 0.2 eV, at the same time the ^1^MLCT states are overestimated by approximately 0.2 eV. This leads to a splitting of the simulated absorption band in the visible region into a low‐lying ILCT and higher lying MLCT bands.

### 2.2 Excited‐State Electron‐Transfer Processes

Scalar‐relativistic TDDFT simulations including SOCs among the low‐lying singlet and triplet states reveal that intersystem crossing in **RuCl** occurs predominantly between strongly coupled ^1^MLCT (e.g., S_10_, S_11_, and S_13_) and their ^3^MLCT counterparts (up to almost 300 cm^−1^). The SOCs between the ^1^ILCT state (S_2_) and the adjacent ^3^ILCT (and ^3^LLCT) states are one or even two orders of magnitude smaller. Notably, this result is consistent along all four equilibrium structures studied herein (see Tables S11–S14 for S_0_, ^3^MLCT, ^3^ILCT, and ^3^LLCT structures) and is in full agreement with our recently studied transition metal complexes incorporating an organic donor moiety.^[^ ^5^, ^6^, ^12^ ^]^ Thus, we focus our investigation on the excited‐state relaxation processes within the triplet manifold starting from the lowest‐lying ^3^MLCT state and subsequent relaxation processes associated with the lowest‐lying ^3^ILCT and ^3^LLCT states (T_1_–T_3_). Thereby, our approach aims to unravel the interaction between the ^3^ILCT and the ^3^LLCT states and to elucidate the previously observed remote‐control concept of the underlying electronic couplings.^[^ ^5e^ ^]^

Initially, the structures of the three triplet states of interest were optimized. Our simulations reveal only a minor structural rearrangement of the **Ru(tpy)_2_** moiety upon relaxation into the ^3^MLCT state equilibrium geometry, as well as in the case of the ^3^ILCT and ^3^LLCT geometries, compared to the ^1^GS structure (Tables S3–S5). Meanwhile, the structural relaxation of ^3^ILCT and ^3^LLCT primarily involves the PTZ moiety, as illustrated in Table S2. This is ascribed to the photooxidation of the PTZ group, resulting in a partial planarization in the vicinity of the nitrogen atom due to the increase of sp^2^ (and decrease of sp^3^) character. Additionally, the structural equilibration of the ^3^ILCT state is primarily associated with the planarization along the PTZ‐tpy dihedral angle from 27.5° in the ground state to 12.2° in the ^3^ILCT geometry, see Table S1. All optimized equilibrium structures of **RuCl** are accessible via the open repository Zenodo.^[^ ^13^ ^]^

**Figure 4:**
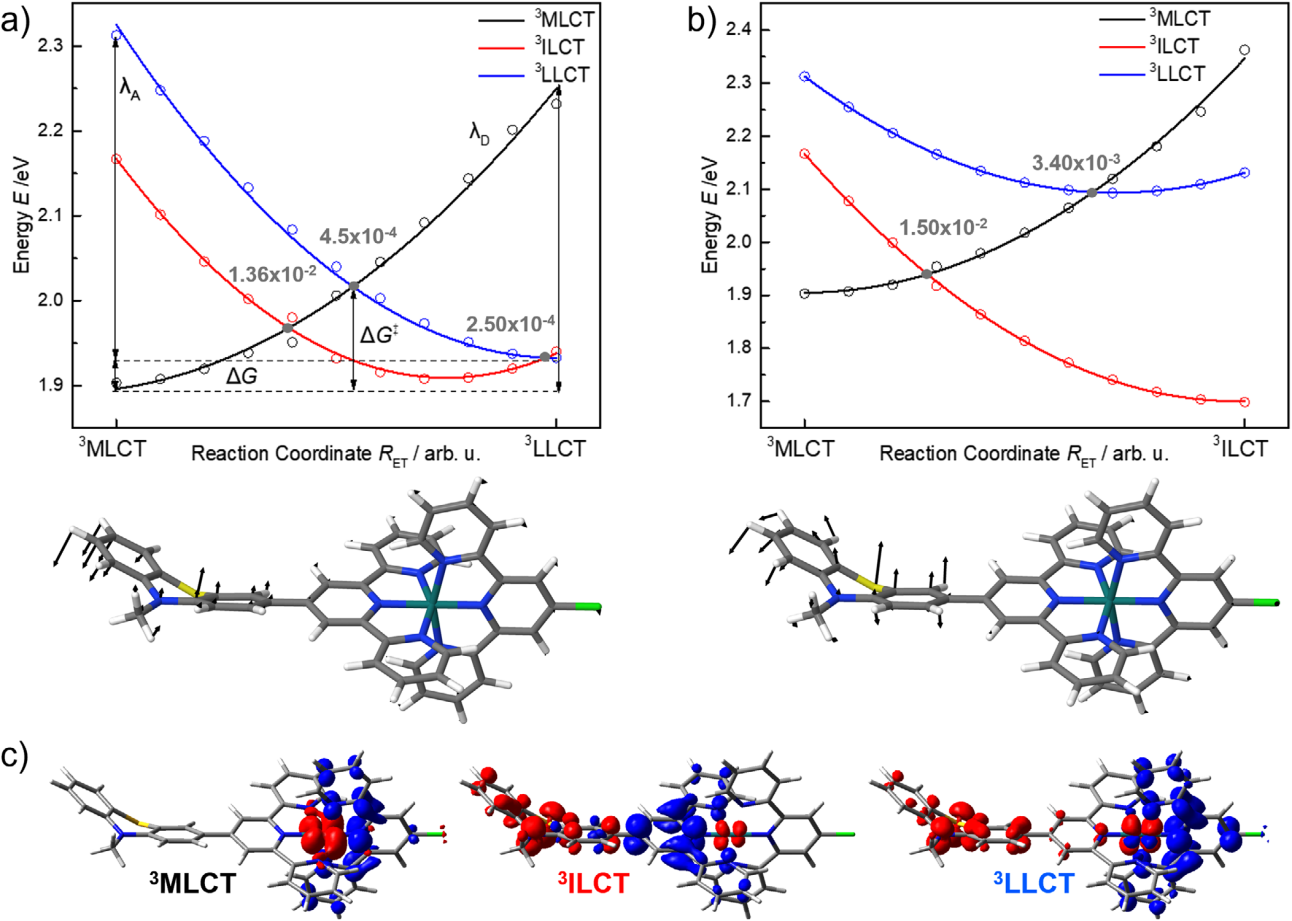
(a) ^3^MLCT and ^3^LLCT pathway connecting the respective relaxed equilibrium structures for **RuCl**, while the LIIC in (b) connects the ^3^MLCT and ^3^ILCT equilibrium structures; see displacement vectors (bottom). Calculated diabatic potential energy curves of the ^3^MLCT donor state (D; black), the ^3^ILCT acceptor state (A; red) and the ^3^LLCT acceptor state (A; blue) along *R*_ET_ for **RuCl**. Driving force (Δ*G*) and reorganization energies (*λ*_D_ and *λ*_A_) are indicated in (a). The solid gray dots denote the crossing point between two states. A quadratic polynomial, $E\;\left(R_{\mathrm{ET}}\right)=a{\left(R_{\mathrm{ET}}-R_{0}\right)}^{2}+E_{0}$, was fitted to the respective data sets. (c) Electronic characters for states of interest are visualized by charge density differences (CDDs); charge transfer occurs from red to blue.

Hereafter, the ET kinetics between the ^3^MLCT donor state and the ^3^ILCT and ^3^LLCT acceptor states are evaluated with semiclassical Marcus theory. Notably, this ^3^MLCT state localizes the excited electron in the lowest energy $\pi_{\mathrm{tpy}}^{*}$ orbital of the tpy‐Cl ligand (Figure 4c). The respective ^3^MLCT transition involving the PTZ‐tpy‐Ru unit is found at higher energy due to the electron donating character of the PTZ group. In the followingsection, we will focus our computational investigation exclusively on the lowest energy ^3^MLCT state (Ru‐tpy‐Cl). In the case of the ^3^MLCT‐^3^LLCT channel (shown in Figure 4a,c), a slightly endergonic driving force of 0.03 eV is obtained. Reorganization energies of 0.33 and 0.38 eV for the ^3^MLCT donor and the ^3^LLCT acceptor state (*λ*_D_ and *λ*_A_, respectively) yield an average reorganization energy (*λ*_AVG_) of 0.36 eV; see Table 1. The nearly identical reorganization energies show that the two potential energy curves (PECs; ^3^MLCT, and ^3^ILCT) exhibit almost identical curvatures, indicating that the applied linear‐interpolated internal coordinate (LIIC) is appropriate within the Marcus picture. In addition to the driving force (Δ*G*) and the reorganization energy (*λ*), the ET process is governed by the electronic communication between the involved diabatic states, commonly denoted as the electronic coupling (*V*_DA_). Our previous work indicated that different substituents (Figure 1) offered significant control on *V*_DA_ ranging from 4.5 × 10^−4^ to 11.5 × 10^−4 ^eV, while the small magnitude of the electronic coupling between the ^3^MLCT and ^3^LLCT state indicates an inefficient communication between these states.^[^ ^5e^ ^]^ Our simulations concerning **RuCl** suggest a very similar situation with a *V*_DA_ value of merely 4.50 × 10^−4^ eV between the ^3^MLCT and ^3^LLCT state. Notably, all three applied methods to assess *V*_DA_, namely the generalized Mulliken–Hush (GMH) method,^[^ ^14^ ^]^ the fragment charge difference (FCD) approach and minimum energy splitting method, yield the same results, see Table S10 for details. In agreement with our previous investigation, this small *V*_DA_ value between the ^3^MLCT and the ^3^LLCT state is attributed to the large distance between the involved redox centers as well as the almost orthogonal orientation among PTZ‐tpy and the tpy‐Cl ligand spheres. Finally, the rate constant for the ET from the ^3^MLCT state to the ^3^LLCT state was calculated within the Marcus picture by means of Δ*G*, *λ*_AVG_ and *V*_DA_. Based on the weak coupling and the slightly endothermic driving force, a comparably slow ET process with a rate of 6.91 × 10^8^ s^−1^ is predicted (Table 1).

**Table 1:** TDDFT‐simulated driving forces (Δ*G*), activation energies (Δ*G* ^ǂ^), reorganization energies (top to bottom: *λ*_D_, *λ*_A_, and *λ*_AVG_), electronic couplings (*V*_DA_), and rate constants (*k*_ET_) for **RuCl** as obtained within the semiclassical Marus picture.

^3^MLCT (D)–^3^LLCT (A)					^3^MLCT (D)–^3^ILCT (A)				
Δ*G* [eV]	Δ*G^ǂ^* [eV]	*V*_DA_ [eV]	*λ_i_* [eV]	*k*_ET,_ *_i_* [s^−1^]	Δ*G* [eV]	Δ*G^ǂ^* [eV]	*V*_DA_ [eV]	*λ_i_* [eV]	*k*_ET,_ *_i_* [s^−1^]
0.03	0.15	4.50 × 10^−4^	0.33	9.44 × 10^8^	−0.20	0.04	1.50 × 10^−2^	0.46	1.28 × 10^12^
			0.38	5.07 × 10^8^				0.47	1.18 × 10^12^
			0.36	6.91 × 10^8^				0.47	1.23 × 10^12^

In an analogous fashion, the ^3^MLCT‐^3^ILCT relaxation pathway was examined as illustrated in Figure 4b,c. In the case of the ^3^MLCT‐^3^ILCT channel, an exergonic Δ*G* of −0.20 eV was calculated, again almost identical reorganization energies of 0.46 (^3^MLCT) and 0.47 eV (^3^ILCT) were predicted at the TDDFT level of theory along the reaction coordinate (*R*_ET_). The electronic coupling, calculated using the same three methods, was determined within the crossing region of the diabatic ^3^MLCT and ^3^ILCT states. For this particular pair of redox states, *V*_DA_ was obtained as 1.50 × 10^−2^ eV, which is two orders of magnitude larger than the ^3^MLCT‐^3^LLCT coupling. This sizeable *V*_DA_ is a result of the favorable orientation and the short distance of the PTZ‐tpy‐Ru units. Consequently, the ET kinetics along the ^3^MLCT‐^3^ILCT pathway (∼10^12^ s^−1^, Table 1) are roughly four orders of magnitude faster than the ET kinetics along the previously discussed ^3^MLCT‐^3^LLCT channel.

In the present study, we have extended our previously introduced simplified two‐state picture to a three‐state picture, which involves the discussed ^3^MLCT, ^3^ILCT, and ^3^LLCT states along the two *R*_ET_ coordinates, as shown in Figure 4. Thus, we also obtained electronic couplings between all three states. In the case of the ^3^MLCT‐^3^LLCT channel as shown in Figure 4a, also the diabatic PECs of the ^3^MLCT and ^3^ILCT states cross along the *R*_ET_ – associated with a barrier of approximately 0.08 eV. The electronic coupling obtained in the vicinity of this crossing point (1.36 × 10^−2^ eV) is almost identical, yet slightly smaller in comparison to the respective *V*_DA_ along the ^3^MLCT‐^3^ILCT coordinate (see Table 1). Furthermore, a barrierless transfer between the ^3^LLCT and ^3^ILCT state is predicted in the vicinity of the relaxed ^3^LLCT structure. However, as in the case of the ^3^MLCT‐^3^LLCT scenario, a very weak coupling of 2.5 × 10^−4^ eV is calculated between ^3^LLCT and ^3^ILCT (Figure 4a), which suggests an inefficient population transfer, even though this ET is in the barrierless Marcus regime. Along the second reaction coordinate (^3^MLCT‐^3^ILCT, Figure 4b), we predict a slightly stronger electronic communication between the ^3^MLCT and the ^3^LLCT state (3.4 × 10^−3^ eV, Figure 4b). However, such ET is associated with an activation energy of 0.2 eV, which renders the respective process unlikely. In addition, efficient and barrierless back ET is expected along this coordinate. The PECs of the ^3^ILCT and ^3^LLCT states do not cross within the investigated conformer space (Figure 4b).

**Figure 5:**
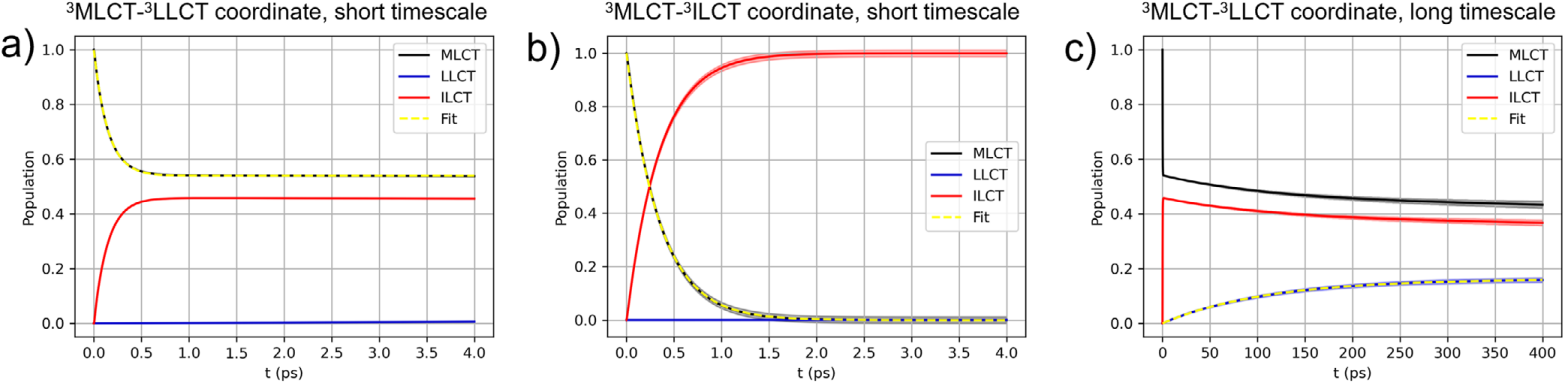
Population densities of ^3^MLCT (black), ^3^LLCT (blue), and ^3^ILCT (red) states according to dissipative quantum dynamics starting from the equilibrated ^3^MLCT state; yellow dashed lines depict the fits used to determine reaction rate constants. Fast population transfer within initial 4 ps. (a) Along the ^3^MLCT‐^3^LLCT coordinate, and (b) along the ^3^MLCT‐^3^ILCT coordinate. (c) Slow population transfer within 400 ps along the ^3^MLCT‐^3^LLCT coordinate.

To obtain a more detailed picture of the photoinduced ET processes between the three states of interest as well as to evaluate potential interference effects, both quantum dynamical simulations and fs transient absorption (TA) spectroscopy were applied. Based on the three‐state picture in the performed DQD, a fast population transfer from the ^3^MLCT to the ^3^ILCT state is predicted within the first 4 ps. This result is independent of the considered reaction coordinate, Figure 4a versus Figure 4b, resulting in similar rate constants of 2.93 × 10^12^ and 6.85 × 10^12^ s^−1^, respectively. Also, the influence of excess energy, that is, considering a vibrationally “hot MLCT” state as initial state in the simulations rather than a vibrationally relaxed MLCT, does not alter the results significantly (and yields rates of 2.87 × 10^12^ and 8.24 × 10^12^ s^−1^). Notably, a complete population transfer from the ^3^MLCT to the ^3^ILCT state is obtained along the ^3^MLCT‐^3^ILCT coordinate (see Figure 5b), while an incomplete transfer is observed along the ^3^MLCT‐^3^LLCT coordinate resulting in 54% ^3^MLCT population and 45% ^3^ILCT population after 4 ps (see Figure 5a). This roughly 1:1 equilibration is in line with the small (unfavorable) driving force of approximately 5.90 × 10^−3^ eV. Again, almost identical results are obtained for the “hot MLCT” scenario as shown in Figure S16. Therefore, no population of the ^3^LLCT state is predicted based on the investigated parameter space within the short time scale (first 4 ps).

Subsequently, we extended our DQD simulations from the short timescale (4 ps, Figure 5a,b) to 400 ps (Figure 5c) to investigate a potential slow population transfer to the ^3^LLCT state on a longer timescale. Here, we focus exclusively on the ^3^MLCT‐^3^LLCT coordinate, which in contrast to the ^3^MLCT‐^3^ILCT coordinate, features a low‐lying and energetically accessible ^3^LLCT state. Indeed, within the 400 ps timeframe, a slow and incomplete population transfer both from the ^3^MLCT as well as from the ^3^ILCT state towards the ^3^LLCT is observed, see Figure 5c). The ^3^MLCT population decreases from 54% at 4 ps to 43% at 400 ps, likewise, the ^3^ILCT population decreases from 45% (4 ps) to 37% after 400 ps. In consequence, the population of the ^3^LLCT increases slowly (from 0.3%) to 16% at the long timescale of 400 ps, which translates to an ET rate of 9.10 × 10^9^ s^−1^, see Table 2. Therefore, our computational studies clearly show that the majority of population transfer occurs between the strongly coupled ^3^MLCT‐^3^ILCT states, while a maximum of 16% ^3^LLCT population was predicted by the performed DQDs simulation along the ^3^MLCT‐^3^LLCT coordinate. Notably, both ^3^ILCT and ^3^LLCT geometries are similar and differ significantly from the ^1^GS geometry and the ^3^MLCT structure, which is mainly related to partial planarization in the vicinity of the nitrogen atom of PTZ donor upon (photo‐)oxidation, see Table S2 and ref. [13]. This structural similarity between the two acceptor states in combination with the maximum population transfer to the ^3^LLCT state already achieved at early timescales (at 200 ps see Figure 5c) indicates the involvement of the ^3^LLCT acceptor state in the excited state relaxation of **RuCl**.

**Table 2:** Electron transfer rates (*k*_ET_ in s^−1^) determined via fitting the dissipative quantum dynamics simulations starting from either the equilibrated or “hot MLCT” state along the ^3^MLCT‐^3^LLCT and ^3^MLCT‐^3^ILCT coordinates.

	Short Timescale [4 ps]		Long Timescale [400 ps]	
	^3^MLCT‐^3^LLCT	^3^MLCT‐^3^ILCT	^3^MLCT‐^3^LLCT	
Starting Condition	*k*_ET_, ^3^MLCT → ^3^ILCT	*k*_ET_, ^3^MLCT → ^3^ILCT	*k*_ET_, ^3^MLCT + ^3^ILCT → ^3^LLCT	*k*_ET_, ^3^MLCT → ^3^CS (^3^LLCT + ^3^ILCT)
Eq. MLCT	6.85 × 10^12^	2.93 × 10^12^	9.10 × 10^9^	1.25 × 10^10^
“Hot MLCT”	8.24 × 10^12^	2.87 × 10^12^	9.18 × 10^9^	1.29 × 10^10^

As stated in the introduction, transient absorption spectroscopy cannot differentiate between the spectral signatures of the ^3^ILCT and the ^3^LLCT states. Therefore, and in order to enhance the comparability with the experimental results as obtained by temperature‐dependent pump‐probe spectroscopy, we obtained a rate constant for population transfer from the ^3^MLCT state to a ^3^CS, which consists of the joint contributions of the ^3^ILCT as well as the ^3^LLCT population density as obtained along the ^3^MLCT‐^3^LLCT coordinate (up to 400 ps, long timescale). A first order equilibration reaction (see Equation 7) was fitted to the obtained ^3^CS states population density, obtaining reaction rates of 1.25 × 10^10^ s^−1^ and 1.29 × 10^10^ s^−1^ for the equilibrated and the “hot MLCT” states, respectively. Notably, these ET rates are approximately two orders of magnitude slower than the previously discussed ^3^MLCT to ^3^ILCT population transfer and one order of magnitude faster than the ^3^MLCT to ^3^LLCT electron transfer, see Table 2.

**Figure 6:**
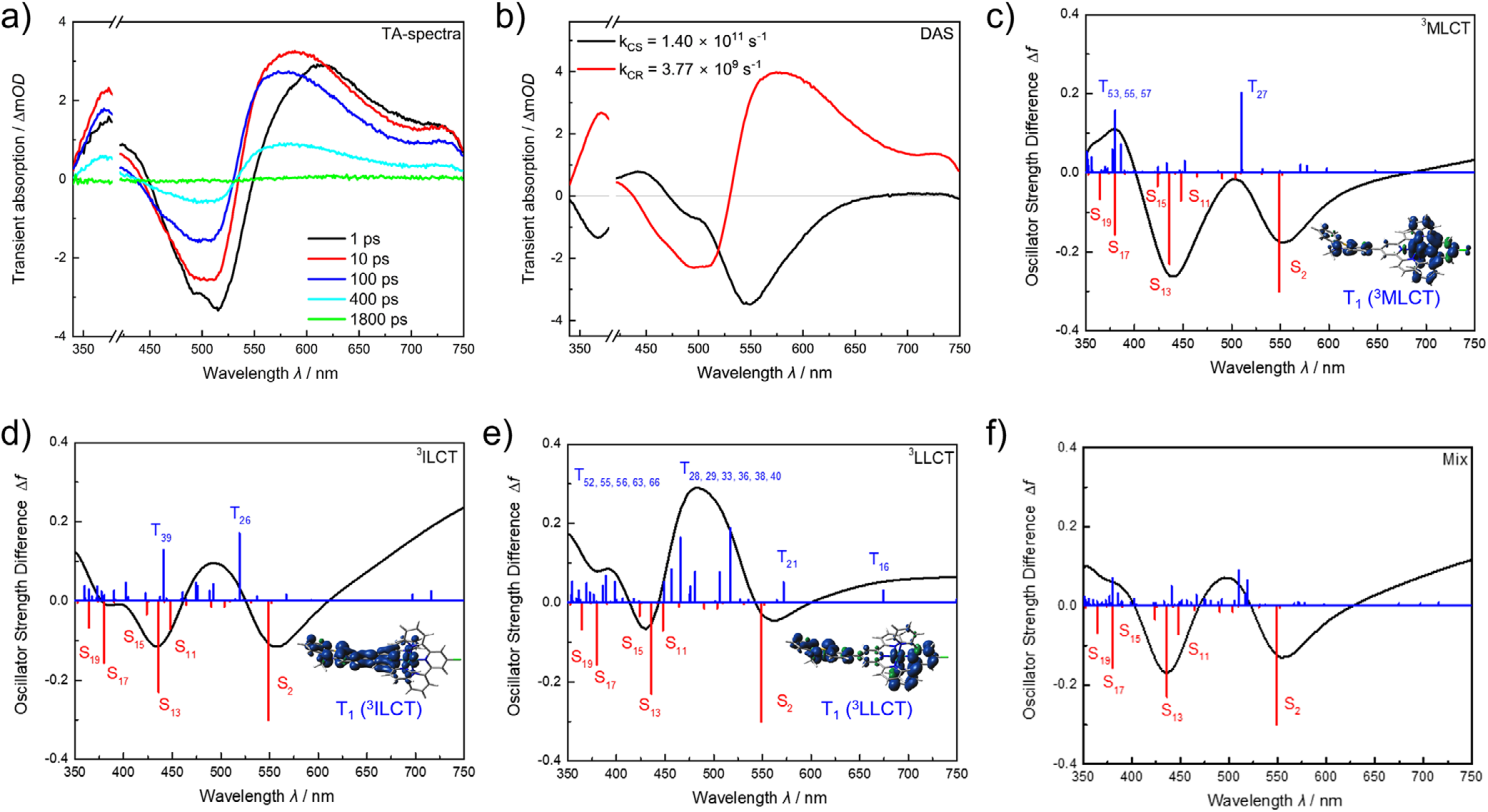
(a) Femtosecond transient absorption spectra at various delay times, recorded at 300 K. (b) Decay associated spectra resulting from global fitting with two finite components (referring to *k*_CS_: the decay of the relaxed ^3^MLCT state towards the ^3^CS states and *k*_CR_: the subsequent decay of the ^3^CS states back to the^1^GS state) of the TA‐data recorded at 300 K. (c–e) Simulated transient absorption spectra within ^3^MLCT, ^3^ILCT, and ^3^LLCT equilibrium geometries, respectively, and obtained at the TDDFT level of theory; spin densities are shown to visualize the nature of the respective triplet ground state species. (f) Simulated spectrum including excited‐state absorption contributions from ^3^MLCT (43%), ^3^ILCT (37%), and ^3^LLCT (16%) states.

In addition to the theoretical modelling of the excited‐state branching dynamics in **RuCl**, we also applied temperature‐dependent pump‐probe spectroscopy to experimentally probe the excited state relaxation channels. The evolution of the fs TA signal at 300 K is shown in Figure 6a. According to previous spectroelectrochemical studies^[^ ^5b^ ^]^ of PTZ and fs TA analysis of other PTZ containing Ru(II)‐bipyridyl systems,^[^ ^5b–d^ ^]^ the excited‐state absorption (ESA) between 350 nm and the (cut out) scattering range of the pump pulse of 380 nm, as well as the ESA band above 580 nm, originates from the oxidized PTZ radical cation (PTZ^•+^). The ground‐state bleach (GSB) covers the wavelength region of the ^1^MLCT/^1^ILCT absorption around 500 nm. By the end of the experimentally observed delay time the transient absorption signal essentially decayed to zero, with full ground‐state recovery on a nanosecond timescale.

In order to assign the individual electronic transitions underlying the ESA and GSB bands, we performed TDDFT simulations with the optimized ^3^MLCT, ^3^ILCT, and ^3^LLCT species. Thereby, the ESA was modelled by spin and dipole‐allowed triplet‐triplet transitions as obtained with the respective optimized triplet species. Contributions to the GSB are accounted for by means of the singlet‐singlet transitions within the Franck–Condon geometry. In the case of the initially populated ^3^MLCT intermediate, the simulated TA spectrum (Figure 6c and Table S7) reveals a strong ESA at 893 nm associated with a weakly dipole‐allowed LMCT transition into T_10_ (see Figure S18a) as well as a strongly dipole‐allowed ILCT at 510 nm (into T_27_). We relate these two transitions to the broad ESA feature extending from approximately 550 to 750 nm as observed at early decay times (1–10 ps). According to the DQD simulations, fast population transfer from the ^3^MLCT to the ^3^ILCT follows. The ESA of the ^3^ILCT species as obtained at the TDDFT level of theory is dominated by two ^3^ILCT transitions ($\pi_{\mathrm{PTZ}}\to \pi_{\mathrm{tpy}}^{*}$, into T_11_, T_26_) at 770 and 519 nm as well as into the T_39_ at 441 nm, which is of ^3^MLCT character involving both tpy ligands; see Figure 6d and Table S8 for details. These computational findings reflect the blue‐shift of the measured ESA within the first 100 ps. As shown in Figure 5c, the ^3^LLCT state is accessible at longer delay times. Notably, this ^3^LLCT absorbs only weakly in the spectral range of interest according to our simulations; see Figure 6c and Table S9 for details. Thus, the decline of ESA at longer delay times can be correlated with the population transfer from the ^3^MLCT and ^3^ILCT states to the weakly coupled ^3^LLCT state.

Thus, the combination of fs TA spectroscopy and theoretical simulations suggests that population from the initially populated ^3^MLCT state (accessible upon ISC) is partially transferred within the first few ps to the ^3^ILCT state. At delay times on the order of few hundreds of ps, excited‐state population is partially transferred to the ^3^LLCT state. Finally, the TA spectrum recorded at 400 ps can be estimated as consisting of 54% ^3^MLCT, 37% ^3^ILCT, and 16% ^3^LLCT excitation—as obtained by DQD (Figure 6f).

Finally, a kinetic model was constructed based on the temperature‐dependent fs TA data. The model consists of two parallel monoexponentially decay processes. KiMoPack^[^ ^15^ ^]^ calculates concentration‐time profiles, decay‐associated spectra (DAS), and the rate for each decay compound included in the model. Figure 6b displays the respective DAS for the experimental data shown in Figure 6a. The first component of the DAS describes the increase of the PTZ^•+^ absorption features. Consequently, the corresponding kinetic rate constant, *k*_CS_ (1.40 × 10^11^ s^−1^, see Figure 6b and Table 3), is correlated with charge separation, that is, the formation of the CS ^3^ILCT or ^3^LLCT states. However, a distinction between ^3^ILCT and ^3^LLCT states is impossible based on experimental data. Thus, we consider that both relaxation channels contribute to the formation of the PTZ^•+^ absorption and refer to this as ^3^CS pathway. The second decay component *k*_CR_ (3.77 × 10^9^ s^−1^, see Figure 6b and Table 3) represents an effective repopulation of the ^1^GS through charge recombination (CR) of the ^3^ILCT and ^3^LLCT states. The respective DAS of *k*_CR_ also resembles the DAS of the CR process of the unsubstituted **RuH** in dichloromethane.^[^ ^5b^ ^]^

**Table 3:** Driving forces (Δ*G*), energy barrier (Δ*G^ǂ^*), reorganization energies *(λ*), electronic couplings (*V*_DA_), rate constants (*k*_ET_), that is, for charge separation (*k*_CS_), and charge recombination (*k*_CR_), as obtained for **RuCl** from electrochemistry and transient absorption spectroscopy at 300 K.

	*k*_ET_ [s^−1^]	Δ*G* ^ǂ^ / eV	Δ*G* ^0^ eV	λ / eV	*V*_DA_ / eV
^3^MLCT‐^3^CS	1.40 × 10^11^	8.73 × 10^−2^	−0.15	0.61	3.20 × 10^−3^
^3^CS‐^1^GS	3.77 × 10^9^	7.91 × 10^−2^	−1.92	1.28	5.37 × 10^−4^

A Marcus analysis of the kinetic rate constants in Figure S15 reveals a linear relation and, thus allows for calculation of the activation barriers for the charge separation $\Delta G_{CS}^{‡}$ (87.3 meV) and charge recombination $\Delta G_{CR}^{‡}$ (79.1 meV) by linear regression.^[^ ^16^ ^]^ In the case of calculating the respective experimental electronic coupling elements *V*_DA_ via the semiclassical Marcus expression (see Equation 4 in the computational details), the reorganization energy and the driving force are required, which are determined by cyclic voltammetry (CV, see Supporting Information for details). The halfstep‐potentials for reduction and oxidation of **RuCl** were used to calculate the standard free charge‐recombination enthalpy $\Delta G_{CR}^{0}$ for the ET from the reduced ligand tpy^•−^ radical anion to fill the hole in the oxidized PTZ^•+^ radical cation donor, using Equation (1).^[^ ^5c^ ^]^ With *e* as elementary charge, $\varepsilon_{0}$ is the dielectric constant in vacuum, $\varepsilon_{S}$ is the relative permittivity of the medium ($\varepsilon_{S}\left(CH_{3}\mathrm{CN}\right)=35.9$) and $r_{\mathrm{DA}}$ is the center‐to‐center distance between the donor and the acceptor moiety, which amounts 6.6 Å for the ^3^ILCT state and 11.9 Å for the ^3^LLCT state. The driving force $\Delta G_{CS}^{0}$ for the related charge‐separation process, starting from the lowest lying ^3^MLCT results from $\Delta G_{\mathrm{CR}}^{0}$ and the emission energy *E*_00_, between the relaxed ^3^MLCT state and the ^1^GS, according to Equation (2).^[^ ^5c^ ^]^ *E*_00_ was estimated from the emission spectrum of **[Ru(tpy)_2_]^2+^** at 77 K (in butyronitrile glass) to be 2.07 eV.^[^ ^5^, ^17^ ^]^ The relation between the driving force $\Delta G^{0}$, reorganization energy *λ* and energy barrier $\Delta G^{‡}$ is given by the Marcus model, as shown in Equation (3):

(1) $$\Delta G_{\mathrm{CR}}^{0}=e\cdot \left[E^{0}\left(A^{0/-}\right)-E^{0}\left(D^{+/0}\right)\right]+\frac{e^{2}}{4\pi \varepsilon_{0}\varepsilon_{S}r_{\mathrm{DA}}}$$

(2) $$\Delta G_{\mathrm{CS}}^{0}=-\Delta G_{\mathrm{CR}}^{0}-E_{00}$$

(3) $$\Delta G^{‡}=0.25\lambda^{-1}{\left(\Delta G^{0}+\lambda \right)}^{2}$$

**Figure 7:**
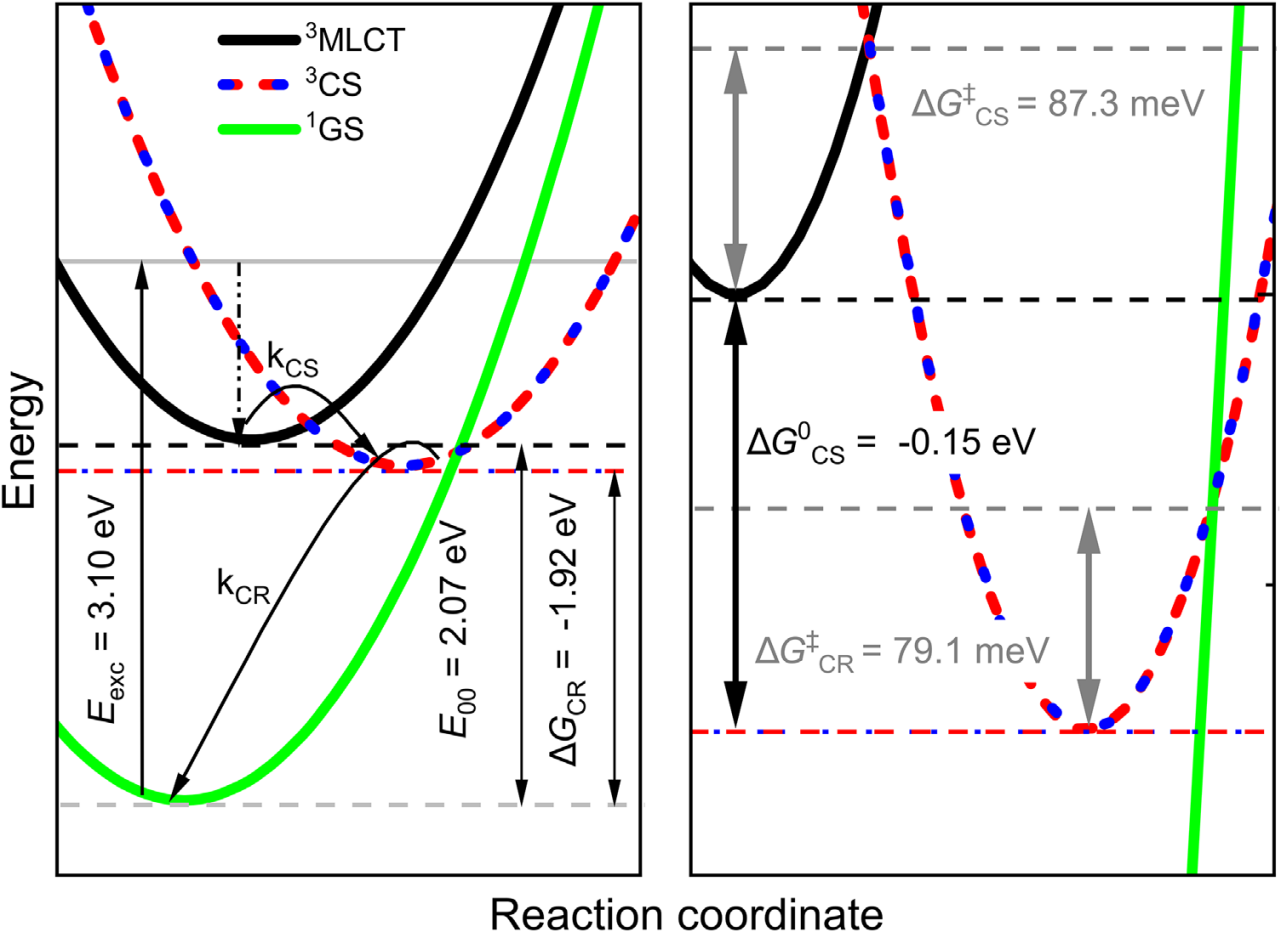
Experimental Marcus picture of the ^3^MLCT, ^3^CS (i.e., combined ^3^ILCT and ^3^LLCT contribution) and ^1^GS potential energy curves, inset shows the enlarged crossing region of the three parabolas.

When estimating $\Delta G_{CR}^{0}$ and $\Delta G_{CS}^{0}$, we assume the spatial separation between the reduced and oxidized molecular units to be the distance from PTZ moiety to the Ru‐center, that is, *r*_DA_ = 9.6 Å. Finally, favorable driving forces of $\Delta G_{\mathrm{CS}}^{0}$= −0.15 eV and $\Delta G_{\mathrm{CR}}^{0}$= −1.92 eV are obtained via Equations (1) and (2). Afterward, reorganization energies, 0.61 and 1.28 eV are obtained when the driving force and energy barrier are inserted into Equation (3). Finally, the electronic couplings are calculated via a semiclassical Marcus model. The global picture containing ^3^MLCT, ^3^CS and ^1^GS PECs is presented in Figure 7.

All key Marcus parameters are listed in Table 3. These experimental results for the driving force, reorganization energy, activation energy, electronic coupling, as well as the rate constant obtained for the electron transfer between the ^3^MLCT and the ^3^CS are in excellent agreement with the theoretical predictions (Tables 1 and 2). As stated above, the experimental data for the ^3^CS channel contains contributions from both ^3^ILCT and ^3^LLCT processes. Therefore, the obtained coupling of 3.20 × 10^−3^ eV and the respective rate constant of 1.40 × 10^11^ s^−1^ can be considered an average of the couplings and rate constants as obtained computationally for the strongly and thus efficient ^3^MLCT‐^3^ILCT population transfer (1.50 × 10^−2^ eV, 1.23 × 10^12^ s^−1^) and the weakly coupled and thus slow ^3^MLCT‐^3^LLCT electron transfer (4.50 × 10^−4^ eV, 6.91 × 10^8^ s^−1^). An improved comparability between the experimental rate constant for the ^3^MLCT‐to‐^3^CS population transfer (1.40 × 10^11^ s^−1^) and our rate constant as obtained by DQD is provided by summation over the ^3^ILCT and ^3^LLCT contributions into a single ^3^CS population density (see Equation 7). This way, a rate constant of ∼1.3 × 10^10^ s^−1^ (Table 2) is obtained, which lies within the range given between the rate of the fast and strongly coupled ^3^MLCT‐^3^ILCT and the rate of the slow and weakly coupled ^3^MLCT‐^3^LLCT processes. Notably, the ^3^LLCT contribution as obtained along the ^3^MLCT‐^3^LLCT coordinate (16% at *t*  =  400 ps) can be considered as upper threshold, while a smaller yet non‐zero ^3^LLCT contribution, e.g. along the ^3^MLCT‐^3^ILCT coordinate, would increase the rate constant even further towards the experimental value.

Our investigation of **RuCl** has allowed us to rationalize the role of competitive excited state relaxation channels, namely those involving ^3^MLCT as well as ^3^ILCT and ^3^LLCT states. The combination of theoretical and experimental spectroscopic techniques, particularly those of DQD and fs TA, respectively have allowed the interplay between these metal and organic based CS states to be examined in greater detail, highlighting the interaction between the states on different timescales as well as the significant influence of the electronic coupling on the electron transfer processes.

## 3 Conclusion

In the present joint spectroscopic‐theoretical study, a Ru(II)‐based photoactive complex (**RuCl**) was investigated with respect to its excited state properties and relaxation cascades. The strategy of combining inorganic and organic chromophores allows the localization of the excited electron density away from metal center, transferring it to the organic chromophore. Thereby, the population of a charge‐separated triplet state on the organic chromophore is achieved. The Franck–Condon photophysics of **RuCl** were thoroughly investigated using TDDFT as well as UV–vis and rR spectroscopy. The jointly applied theoretical and spectroscopic techniques predict a strongly dipole‐allowed low‐energy ^1^ILCT transition and slightly higher lying ^1^MLCT transitions to be accessible in the visible region.

Following initial excitation, ultrafast ISC between ^1^MLCT and ^3^MLCT states occurs and subsequent electron transfer processes between the low‐lying ^3^MLCT state and the energetically adjacent ^3^ILCT and ^3^LLCT states were carefully investigated. The semiclassical Marcus picture of ET using TDDFT, dissipative quantum dynamics and temperature‐dependent pump‐probe spectroscopy were used in conjunction to draw a consistent picture of the excited state relaxation processes involving the three states of interest. In agreement with our previous study,^[^ ^5e^ ^]^ a strongly coupled and thus rapid population transfer from the ^3^MLCT state to the ^3^ILCT acceptor state is observed. This finding reflects the molecular architecture of the push–pull–push structure as the π‐system of the PTZ donor group and the chemically linked acceptor tpy ligand are oriented in the same plane, which results in a strong electronic coupling of 1.5 × 10^−2^ eV. In contrast, weakly coupled (4.50 × 10^−4^ eV) and thus inefficient population transfer along the ^3^MLCT‐^3^LLCT pathway results from the long distance between the involved molecular centers and their almost orthogonal alignment.

However, as determined by DQD simulations and in agreement with the results from fs TA spectroscopy, the rather weakly coupled ^3^LLCT state gains population on the longer timescale of few hundreds of picoseconds from both the ^3^MLCT as well as from the ^3^ILCT state. Notably, the electronic communication of the ^3^LLCT state and the other states can be readily tuned by the introduction of substituents in the periphery of the second terpyridine ligand.^[^ ^5e^ ^]^ These findings reveal a complex interaction between the low‐lying excited states in the present family of Ru(II)‐based transition metal complexes.

Previously, such remote‐control effect on the electron coupling between the ^3^MLCT and ^3^CS states in structurally closely related Ru(II)‐based donor–acceptor–donor systems was introduced by Luo et al., ^[^ ^5d^ ^]^ yet the precise electronic nature of this ^3^CS state was unknown. Based on the present study, we could identify this state to be a combination of a strongly coupled ^3^ILCT acceptor state (major population) and a weakly coupled ^3^LLCT state (minor population). In the context of future applications in photocatalysis, the design strategy to localize the triplet state away from the metal center to create long‐lived charge‐separated states seems highly promising. Of particular interest, is the population transfer to the ^3^LLCT acceptor states as such intermediates provide a large spatial separation of the hole and electron. Therefore, in the follow‐up investigations of our consortium will aim to establish structure‐property relationships that will allow us to tailor the electronic coupling(s) involving the ^3^LLCT target state and thus to increase the efficiency of the population transfer to this long‐distance charge‐separated state.

## 4 Computational Details

All quantum chemical calculations, unless stated otherwise, were performed utilizing the Gaussian 16 program.^[^ ^18^ ^]^ The singlet GS equilibrium structure and electronic properties of **RuCl** were obtained at the density functional level of theory (DFT) using the B3LYP^[^ ^6^, ^12^, ^19^ ^]^ exchange correlation (XC) functional with the def2‐SVP^[^ ^20^ ^]^ basis set, as well as the respective core potentials. Subsequently, a vibrational analysis was carried out within the relaxed ^1^GS structure to verify that a (local) minimum on the 3*N*‐6 dimensional potential energy (hyper‐)surface was obtained. Effects of interaction with the acetonitrile solvent (CH_3_CN: *ε*  =  35.69, *n*  =  1.8609) were taken into account by the solute electron density (SMD) variant of the integral equation formalism of the polarizable continuum model (IEFPCM).^[^ ^21^ ^]^ All calculations were performed including Grimme's D3 dispersion correction with Becke–Johnson damping (D3BJ).^[^ ^22^ ^]^

In order to gain insight into the exited state properties such as excitation energies, transition dipole moments and electronic characters of the 100 lowest excited singlet states as well as of the 50 lowest triplet states within the Franck–Condon region, time‐dependent DFT (TDDFT) calculations were performed using the same XC functional and basis set as utilized in the preceding optimization calculations. Solvent effects on the Franck–Condon photophysics, where only the fast reorganization of the solvent is significant, were addressed by the non‐equilibrium solvation procedure. The above computational protocols have already been successfully applied to elucidate the ground and excited‐state properties of structurally closely related Ru(II) complexes and allows a balanced description of ^3^MLCT, ^3^ILCT, and ^3^LLCT, as well as local intra‐ligand (IL) states.^[^ ^5^, ^23^ ^]^

Furthermore, equilibrium geometries of three specific excited states involved in the subsequent excited state relaxation cascade within the triplet manifold, that is, of the low‐lying ^3^MLCT as well as of the ^3^LLCT and ^3^ILCT states (T_1_, T_2_, and T_3_ within ^1^GS equilibrium structure), were optimized. These states underwent full relaxation at the TDDFT level of theory using our external optimizer pysisyphus,^[^ ^24^ ^]^ which was interfaced with Gaussian 16 for gradient and energy calculations. Wavefunction overlaps^[^ ^25^ ^]^ were utilized to track excited state characters (i.e., ^3^MLCT, ^3^LLCT, and ^3^ILCT) throughout the optimization process. The equilibrium procedure of solvation was applied to all optimizations.

To evaluate scalar‐relativistic effects and their impact on the ISC pathways within the Franck–Condon region, but also within the ^3^MLCT, ^3^LLCT and ^3^ILCT structures, scalar‐relativistic TDDFT calculations were performed utilizing Orca 6.0.1^[^ ^26^ ^]^ using the scalar‐relativistic zeroth‐order regular approximation (SR‐ZORA). DFT and TDDFT calculations were performed using the B3LYP (“Gaussian version”) XC functional;^[^ ^27^ ^]^ the SARC‐ZORA‐TZVP^[^ ^28^ ^]^ basis set was utilized for ruthenium, while all other atoms were described using the respective def2‐TZVP basis sets (with the corresponding SARC/J auxiliary basis set).^[^ ^29^ ^]^ The 20 lowest singlet‐singlet and singlet‐triplet excitations were obtained, while SOCs were obtained at the SR‐ZORA‐TDDFT level of theory. The effects of interaction with CH_3_CN were taken into consideration at the CPCM level of theory.

Furthermore, the transition absorption spectra originating from the three triplet states (i.e., ^3^MLCT, ^3^LLCT and ^3^ILCT) were simulated, where the excited‐state absorption (ESA) was modelled by means of the lowest 200 spin and dipole‐allowed triplet‐triplet transitions obtained within the previously optimized T_1_ equilibrium, respectively. Assuming a 1:1 population of S_0_ and T_1_, the ESAs are given by the triplet–triplet excitation and the ground state bleach (GSB) by the singlet‐singlet excitations within the singlet ground state equilibrium, respectively. This approach allows to reliably model the TA signal upon excitation of the longest wavelength absorption band at long delay times.^[^ ^12^, ^30^ ^]^ Finally, a mixed transient absorption signal was estimated using the individual signal of the three triplet intermediates weighted by their population upon 400 ps (see dissipative quantum dynamics below).

To access the kinetics of photoinduced ET processes involving these three triplet states, semiclassical Marcus theory was applied. According to Marcus theory, ET processes can be modelled as parabolic diabatic potential energy curves (PECs) of the electron donor state (D; ^3^MLCT) and the acceptor state (A; ^3^LLCT, ^3^ILCT) along a given reaction coordinate *R*_ET_. Structural distortion within the donor state—induced by thermal fluctuations of the surrounding bath (e.g., the solvent) – may provide sufficient electronic coupling between D and A to yield a population transfer among the electronic states of interest. Herein, the rate constant, *k*_ET_, for such an ET is given within the Marcus picture by:

(4) $$k_{\mathrm{ET}}=\frac{2\pi }{\hbar }\;|V_{\mathrm{DA}}{|}^{2}{\left(4\pi \lambda k_{B}T\right)}^{-\frac{1}{2}}\exp\left(-\frac{{\left(\Delta G+\lambda \right)}^{2}}{4\lambda k_{B}T}\right).$$

where, *V*_DA_ denotes the electronic coupling between the D and A states at the crossing point of the diabatic PECs, *λ* is the reorganization energy, Δ*G* represents the driving force, that is, the Gibbs free energy, for the ET reaction, *k*_B_ is the Boltzmann constant, and *T* is the absolute temperature.

The ET kinetics for the different pairs of D/A states were described along a linear‐interpolated internal coordinate (LIIC) connecting the optimized equilibrium structures of the D and A states. The diabatic PECs for D and A states were constructed along the LIIC (denoted *R*_ET_) by means of TDDFT single‐point calculations. The electronic couplings were obtained based on the generalized Mulliken–Hush (GMH) method as well as by means of the fragment charge difference (FCD) approach, which are widely applied methods to evaluate the couplings associated with inter‐ and intramolecular ET processes.^[^ ^14^ ^]^ These simulations were performed at the B3LYP/def2‐SVP level of theory using Q‐Chem.^[^ ^31^ ^]^ Solvent effects (CH_3_CN) were taken into account using the conductor‐like polarizable continuum model. More details with respect to the simulation of *V*_DA_ can be found in our recent computational studies.^[^ ^5^, ^32^ ^]^

Finally, the Mask‐Assisted Coarse Graining of Influence Coefficients on Iterative Quasi‐Adiabatic Propagator Path Integrals (MACGIC‐iQUAPI) method was applied.^[^ ^33^ ^]^ Compared to Marcus theory, this method from the family of dissipative quantum dynamics (DQD) simulations captures a detailed system‐bath coupling and propagates wave packages which allows the description of incomplete population transfer processes involving all three states of interest simultaneously (^3^MLCT, ^3^ILCT, and ^3^LLCT). Thereby, an improved accuracy in predicting reaction rates and mechanisms is achieved. Within the MACGIC‐iQUAPI framework, DQD simulations were carried out involving the ^3^MLCT, ^3^ILCT, and ^3^LLCT states along both the ^3^MLCT‐^3^LLCT and ^3^MLCT‐^3^ILCT coordinates. A temperature of 295 K, an ohmic spectral density width of 1000 cm^−1^ and a maximum integration frequency of 32,000 cm^−1^ were used. The number of numerical integration points was set to 64,001 while a propagation time step of approximately 21 fs was utilized. The reorganization energies for each of the three states were averaged. Thus, a reorganization energy of 0.36 eV was assumed along the ^3^MLCT‐^3^LLCT channel, while *λ*_AVG_  =  0.47 eV was used along the ^3^MLCT‐^3^ILCT pathway. The *V*_DA_ values between the states as well as the driving forces and relative positions in equilibrium towards each other were employed as calculated in the Marcus model. The starting point for each propagation was set at the equilibration geometry of the fully populated ^3^MLCT donor state. In an alternative setup, the propagation was initiated in the ^3^MLCT state within the Franck–Condon point (^1^GS geometry). This way, the excited state relaxation processes were studied from a vibronic “hot ^3^MLCT” state. For each simulation, ten propagations were executed and subsequently averaged, accompanied by an error analysis. Reaction rates were derived through fitting procedures conducted along the ^3^MLCT and along the ^3^LLCT state according to Equations (5) and (6).

(5) $$f\left(t\right)=\left(1-M_{\infty }\right)e^{-kt}+M_{\infty }$$

(6) $$f\left(t\right)=L_{\infty }\;*\left(1-e^{-kt}\right)$$

(7) $$f\left(t\right)=\left(CS_{4\mathrm{ps}}-CS_{\infty }\right)e^{-kt}+CS_{\infty }$$

where, f(t) denotes the population density of the respective state at each time step and t the time in ps. $M_{\infty }$ and $L_{\infty }$ are constants and denote the population density in the equilibrated ^3^MLCT and ^3^LLCT states. Equation (7) was solely fitted within the timeframe of 4–400 ps along the ^3^MLCT‐^3^LLCT coordinate. $CS_{4\mathrm{ps}}$ is a constant and denotes the population density of the ^3^CS state at 4 ps. At this reaction time, the fast population transfer from the ^3^MLCT to the ^3^ILCT state is finished and a population transfer between all three states (^3^MLCT, ^3^ILCT and ^3^LLCT) takes place.

## Supporting Information

See the supporting material for details on synthesis of **RuCl**, CV, and fs TA experiments, geometric information, simulated excited‐state properties, excited‐state equilibrium geometries, as well as ET kinetics. All optimized geometries of **RuCl** are available free of charge by Zenodo repository, see ref. [13].

## Conflict of Interests

The authors declare no conflict of interests.

## Supporting information



Supporting information

## Data Availability

The data that support the findings of this study are openly available in Zenodo at https://doi.org/10.5281/zenodo.14060746, reference number 13.
