# Supplementary material for: Excited State Branching Processes in a Ru(II)‐Based Donor–Acceptor–Donor System
Source: Chemistry. 2025 May 3;31(31):e202404671. doi: 10.1002/chem.202404671 (PMC12133645; doi:10.1002/chem.202404671)
Supplement: Supplementary file 1 — Supporting information [file CHEM-31-e202404671-s001.pdf]

Supporting Information

Excited State Branching Processes in a Ru(II)-based Donor-Acceptor-Donor System

Guangjun Yang,[a] Louis Blechschmidt,[a,b] Linda Zedler,[b] Clara Zens,[a] Kamil Witas[c], Maximilian Schmidt,[d] Birgit Esser,[d] Sven Rau[c], Georgina E. Shillito,[a] Benjamin Dietzek-Ivanšić[a,b,e] and Stephan Kupfer[a]*

[a] G. Yang, L. Blechschmidt, Clara Zens, Dr. G. E. Shillito, Dr. S. Kupfer, Prof. Dr. B. Dietzek-Ivanšić
Institute of Physical Chemistry, Friedrich Schiller University Jena,
Helmholtzweg 4, 07743 Jena, Germany
E-mail: stephan.kupfer@uni-jena.de

[b] L. Blechschmidt, Dr. L. Zedler, Prof. Dr. B. Dietzek-Ivanšić
Leibniz Institute of Photonic Technology (IPHT). Department Functional Interfaces,
Albert-Einstein-Straße 9, 07745 Jena, Germany

[c] K. Witas, Prof. Dr. Sven Rau
Institute for Inorganic Chemistry 1, Ulm University,
Ulm 89081, Germany

[d] Dr. M. Schmidt, Prof. Dr. Birgit Esser
Institute of Organic Chemistry II and Advanced Materials, Ulm University
Albert-Einstein-Allee 11, 89081 Ulm, Germany

[e] Prof. Dr. B. Dietzek-Ivanšić (current address)
Leibniz Institute of Surface Engineering e.V. (IOM)
Permoserstraße 15, 04318 Leipzig, Germany

Experimental Details

Synthesis:

PTZ-tpy was synthesized according to literature.[1] All other chemicals were purchased from commercial suppliers and used as received. Dimethylformamide (DMF) and acetonitrile (MeCN) were purchased from Sigma Aldrich (HPLC grade, 99,9%). All other solvents were purchased by VWR in technical grade and distilled prior to use.

[(4′-Cl-tpy)RuCl$_3$]:

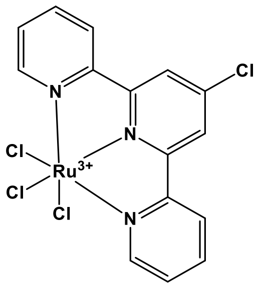

The complex was synthesized according to a literature known procedure.[2]

228 mg of RuCl$_3$ x 3H$_2$O (0.88 mmol) and 234 mg of 4′-chloro-2,2′:6′,2′′-terpyridine (4´-Cl-tpy; 0.88 mmol) were added to 120 mL of absolute EtOH and stirred under reflux for 3 hours. After cooling to room temperature, the formed precipitate was filtered and washed with absolute EtOH and diethyl ether to get 359 mg (0.76 mmol, 84 %) of the product as a dark red powder. The product was used as received.

[(4′-Cl-tpy)Ru(MeCN)$_3$](PF$_6$)$_2$:

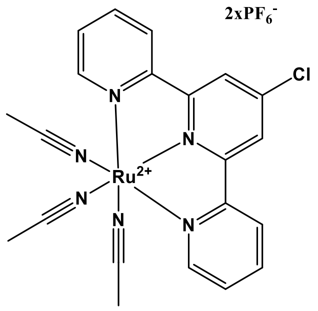

The complex was synthesized according to a literature known procedure.[3]

180 mg (0.38 mmol) [(4′-Cl-tpy)$RuCl_3$] and 200 mg (1.17 mmol) $AgNO_3$ were suspended in 8 mL of MeCN/EtOH/H_2O (6:1:1) and stirred at 80°C for 24 hours, After the reaction solution cooled down to room temperature, the solution was filtered through a pad of celite and rinsed with MeCN. The solution was concentrated under vacuum and slowly added to a concentrated solution of aqueous NH_4PF_6. The formed precipitated was filtered off and washed with water, diethyl ether and dried under air. The crude product was further purified via vapor diffusion crystallization from MeCN and diethyl ether. The formed crystalline powder was filtered and washed with diethyl ether to get 111 mg (0.143 mmol, 38 %) of the product as an orange crystalline powder.

^{1}H-NMR (400 MHz, CD_3CN): δ 8.93 (d, *J* = 5.4 Hz, 2H), 8.50 (s, 2H), 8.41 (d, *J* = 8.0 Hz, 2H), 8.20 (td, *J* = 7.9, 1.5 Hz, 2H), 7.80 – 7.74 (m, 2H), 2.74 (s, 3H) 1.94 (s, 6H overlap with solvent signal).

^{1}H-NMR (400 MHz, $(CD_3)_2SO$): δ 9.06 (s, 2H), 9.02 (d, *J* = 4.7 Hz, 2H), 8.81 (d, *J* = 7.9 Hz, 2H), 8.33 (td, *J* = 7.9, 1.4 Hz, 2H), 7.93 – 7.86 (m, 2H), 2.89 (s, 3H), 2.16 (s, 6H).

^{13}C-NMR (101 MHz, CD_3CN): δ 160.78, 158.60, 155.57, 145.68, 140.17, 129.51, 128.59, 125.44, 124.50, 124.20, 4.74, 4.00.

[(PTZ-tpy)Ru(4′-Cl-tpy)]$(PF_6)_2$ (RuCl):

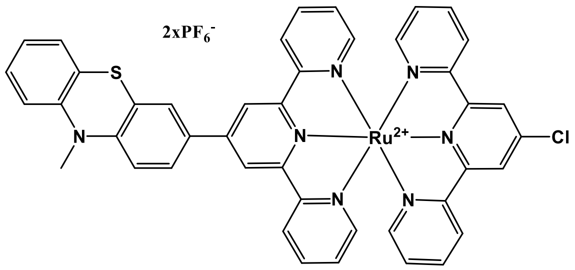

The synthesis was adapted from the literature.[1b, 4]

47.78 mg of [(4′-Cl-tpy)$Ru(MeCN)_3$]$(PF_6)_2$ (0.061 mmol) and 27.7 mg (0.062 mmol) of PTZ-tpy were dissolved in 10 ml of DMF, the solution was degassed with Argon for 20 min and heated to 96°C in a microwave under an argon atmosphere for 2.5 hours. After cooling to room temperature, diethyl ether was added until a precipitate was

formed. The precipitate was filtered and washed with diethyl ether and further purified via column chromatography (silica; MeCN/H_2O/satd. (aq.) KNO_3 solution; 40:4:1). An excess of NH_4PF_6 was added to the fraction containing the product. The solvent was concentrated under vacuum until a precipitation occurred, then water was added to complete the precipitation and the precipitate was filtered, washed with water and the product was rinsed off with MeCN and dried under vacuum to give 26 mg (0.024 mmol, 39%) of the product as a red powder.

^{1}H NMR (400 MHz, CD_3CN) δ 8.96 (s, 2H), 8.84 (s, 2H), 8.64 (d, *J* = 7.9 Hz, 2H), 8.49 (d, *J* = 7.9 Hz, 2H), 8.12 – 8.02 (m, 2H), 7.97 – 7.91 (m, 4H), 7.43 (dd, *J* = 5.6, 0.7 Hz, 2H), 7.36 (dd, *J* = 5.6, 0.7 Hz, 2H), 7.34 – 7.29 (m, 1H), 7.27 – 7.14 (m, 6H), 7.08 – 7.03 (m, 2H), 3.52 (s, 3H).

^{13}C-NMR (101 MHz, CD_3CN): δ 159.18, 158.17, 157.35, 156.26, 153.60, 153.55, 148.87, 148.29, 145.91, 143.62, 139.13, 131.34, 129.13, 128.93, 128.38, 128.29, 128.04, 126.77, 125.86, 125.51, 125.22, 124.99, 124.24, 122.99, 121.62, 116.16, 116.08, 36.26.

HR-MALDI-MS: [M] = [$C_{43}H_{30}ClF_{12}N_7P_2RuS$] = 1103.0299

[M-2PF_6^-]$^+$ calc.: 813.1009; found: 813.1021, [M-2PF_6^-+O]$^+$ calc.: 829.0959; found: 829.0967, [M-2PF_6^-+F^-]$^+$ calc.: 832.0993; found: 832.1006, [M-PF_6^-]$^+$ calc.: 958.0651; found: 958.0666, [M-PF_6^-+O]$^+$ calc.: 974.0600; found: 974.0625, [M] calc.: 1103.0299; found: 1103.0328, [2M-PF_6^-]$^+$ calc.: 2061.0950; found: 2061.1013, [2M-PF_6^-+O]$^+$ calc.: 2077.0899; found: 2077.0984, [2M-PF_6^-+2O]$^+$ calc.: 2093.0849; found: 2093.0985, [3M-PF_6^-+H]$^+$ calc.: 3165.1327; found: 3165.1424, [3M-PF_6^-+2H+O]$^+$ calc.: 3182.1355; found: 3182.1371, [3M-PF_6^-+2O]$^+$ calc.: 3196.1148; found: 3196.1289, [3M-PF_6^-+2H+3O]$^+$ calc.: 3214.1253; found: 3214.1206, [4M-PF_6^-]$^+$ calc.: 4267.1548; found: 4267.2132, [4M-PF_6^-+O+H]$^+$ calc.: 4284.1576; found: 4284.2083, [4M-PF_6^-+2O+H]$^+$ calc.: 4300.1525; found: 4300.2038, [4M-PF_6^-+3O+3H]$^+$ calc.: 4318.1630; found: 4318.1861, [4M-PF_6^-+4O+2H]$^+$ calc.: 4333.1501; found: 4333.1939.

NMR Spectroscopy:

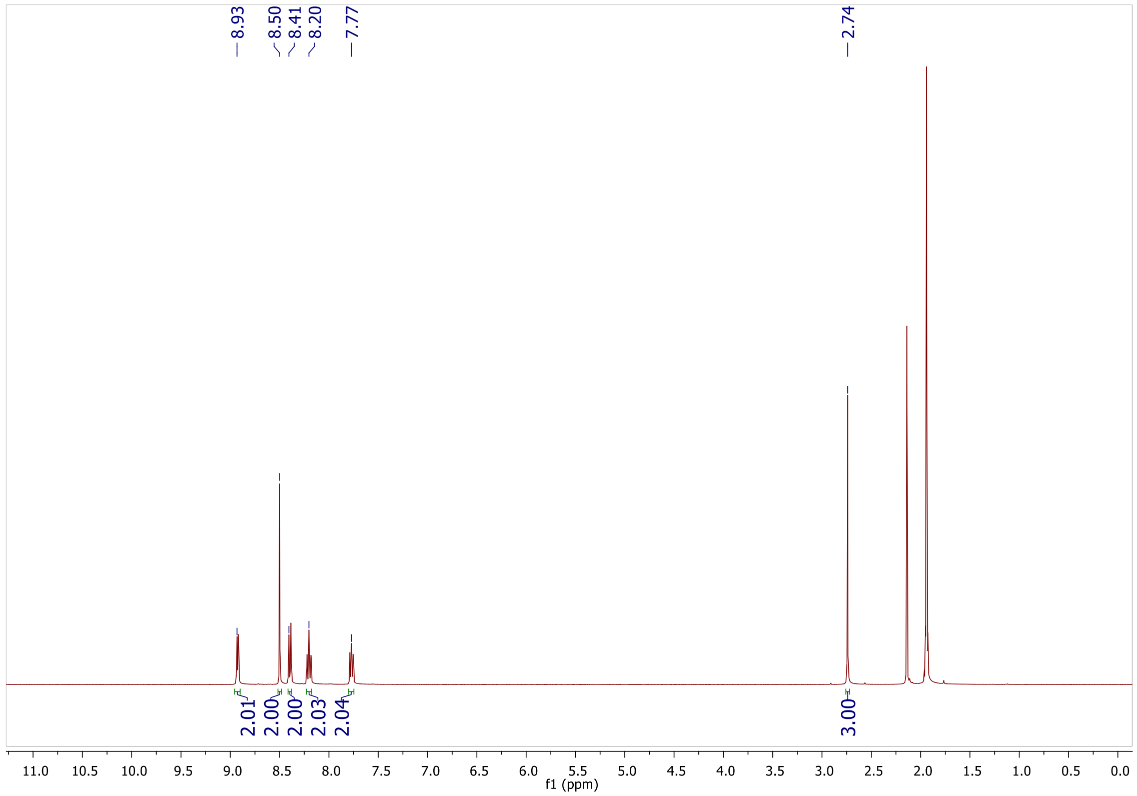

Figure S1 ^{1}H-NMR spectrum of [(4′-Cl-tpy)Ru(MeCN)$_3$](PF$_6$)$_2$ in CD$_3$CN.

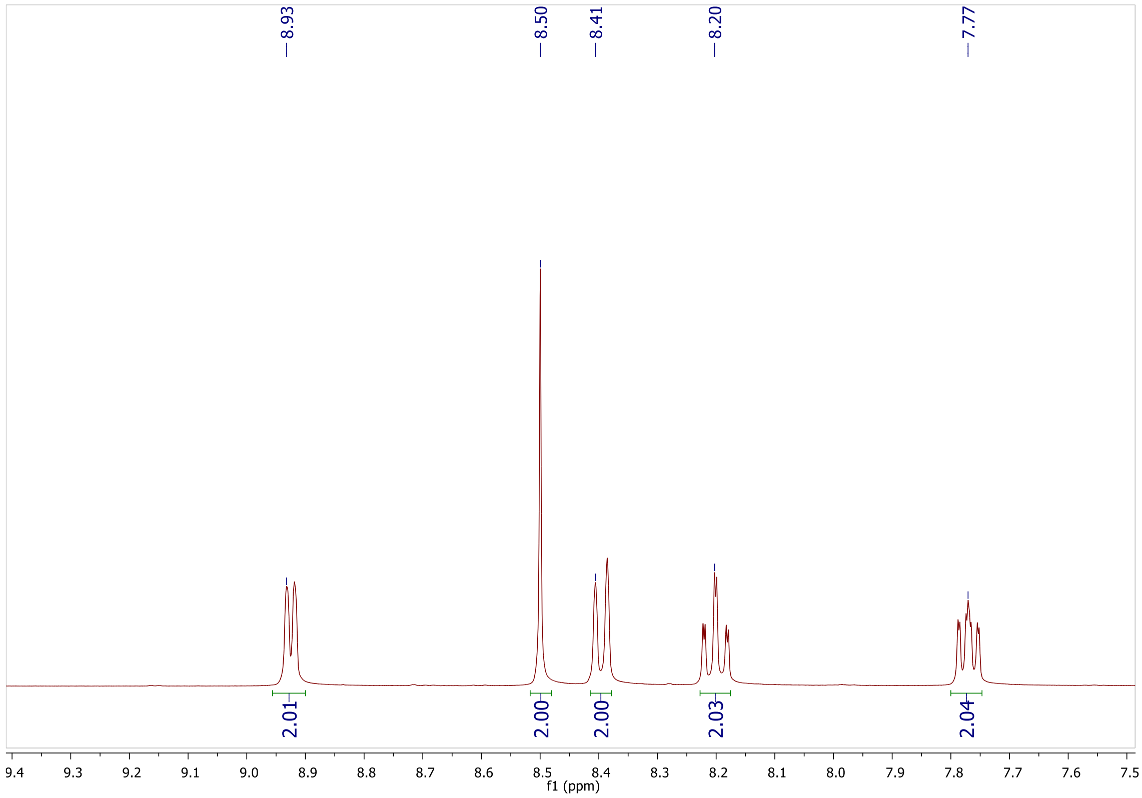

Figure S2 ^{1}H-NMR spectrum of [(4′-Cl-tpy)Ru(MeCN)$_3$](PF$_6$)$_2$ in CD$_3$CN. (aromatic region)

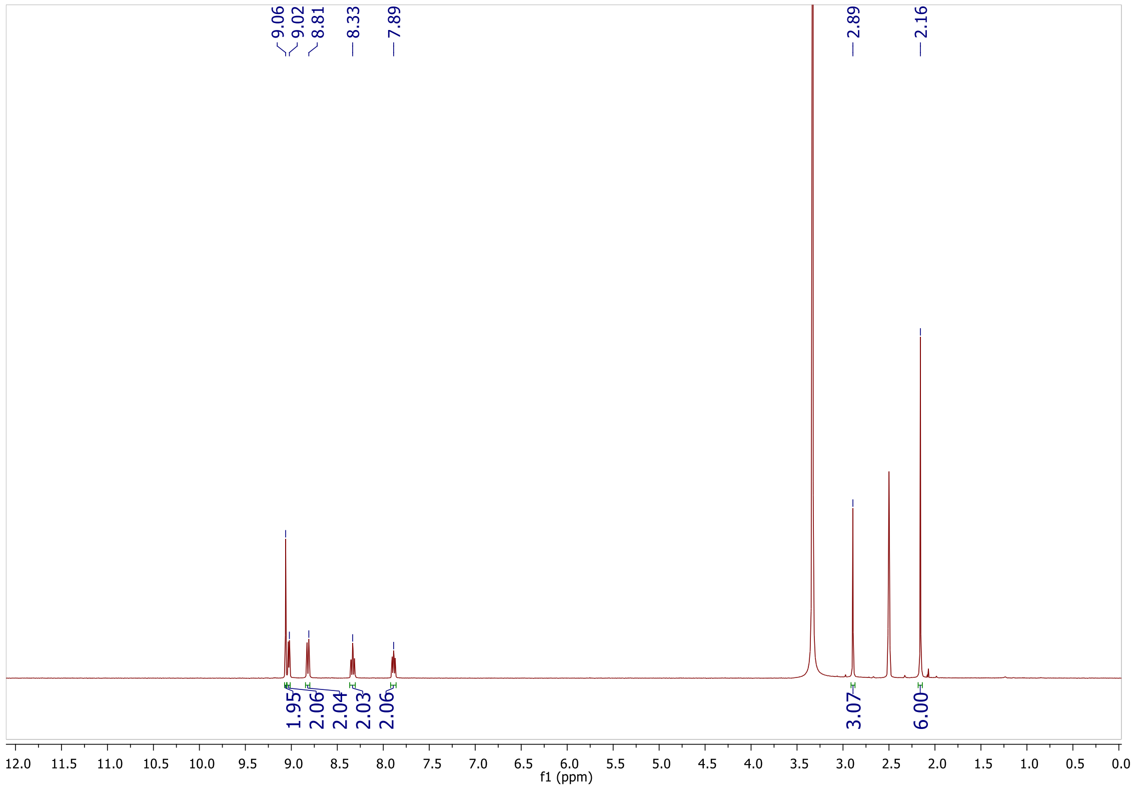

Figure S3 ^{1}H-NMR spectrum of [(4′-Cl-tpy)Ru(MeCN)$_3$](PF$_6$)$_2$ in (CD$_3$)$_2$SO.

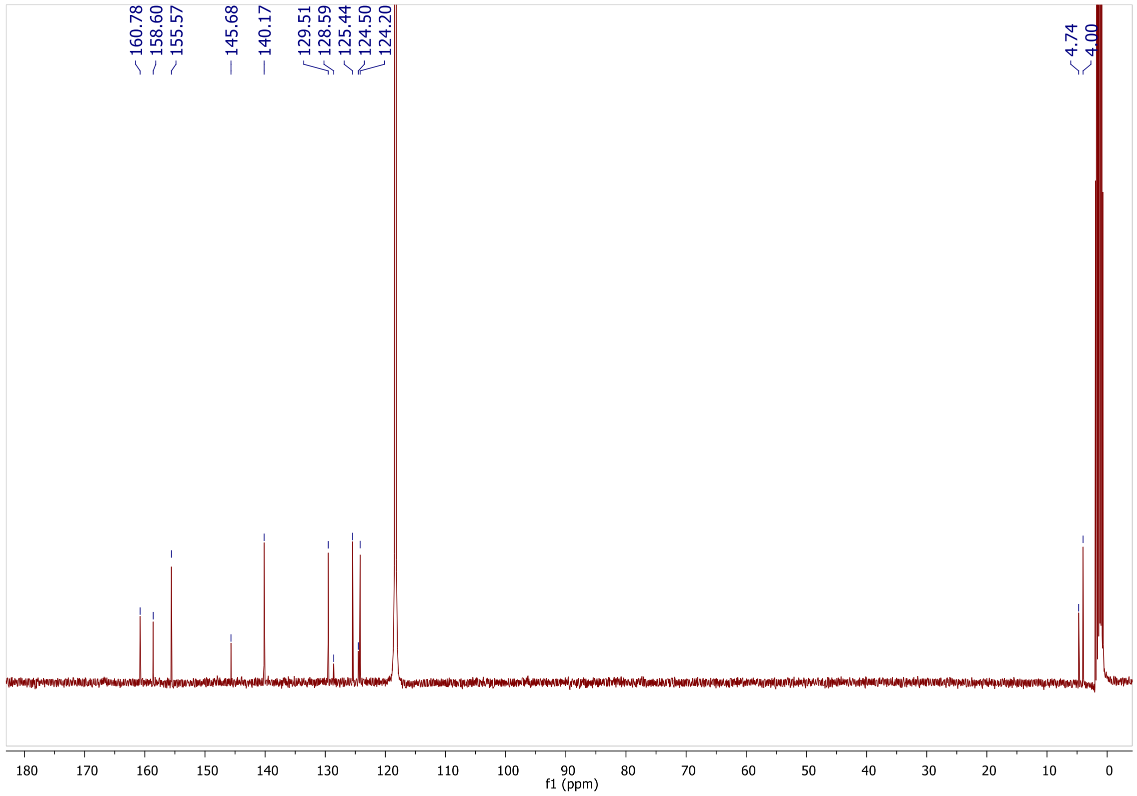

Figure S4 ^{13}C-NMR spectrum of [(4′-Cl-tpy)Ru(MeCN)$_3$](PF$_6$)$_2$ in CD$_3$CN.

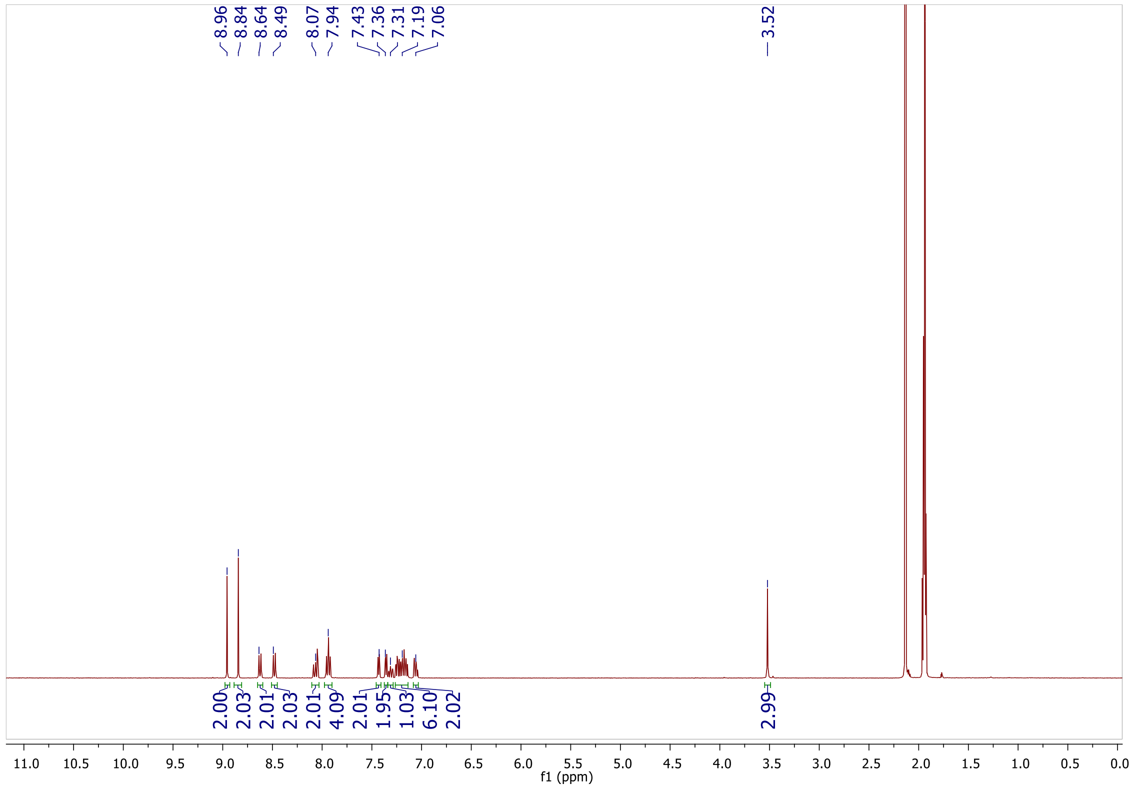

Figure S5 ^{1}H-NMR spectrum of **RuCl** in CD_3CN.

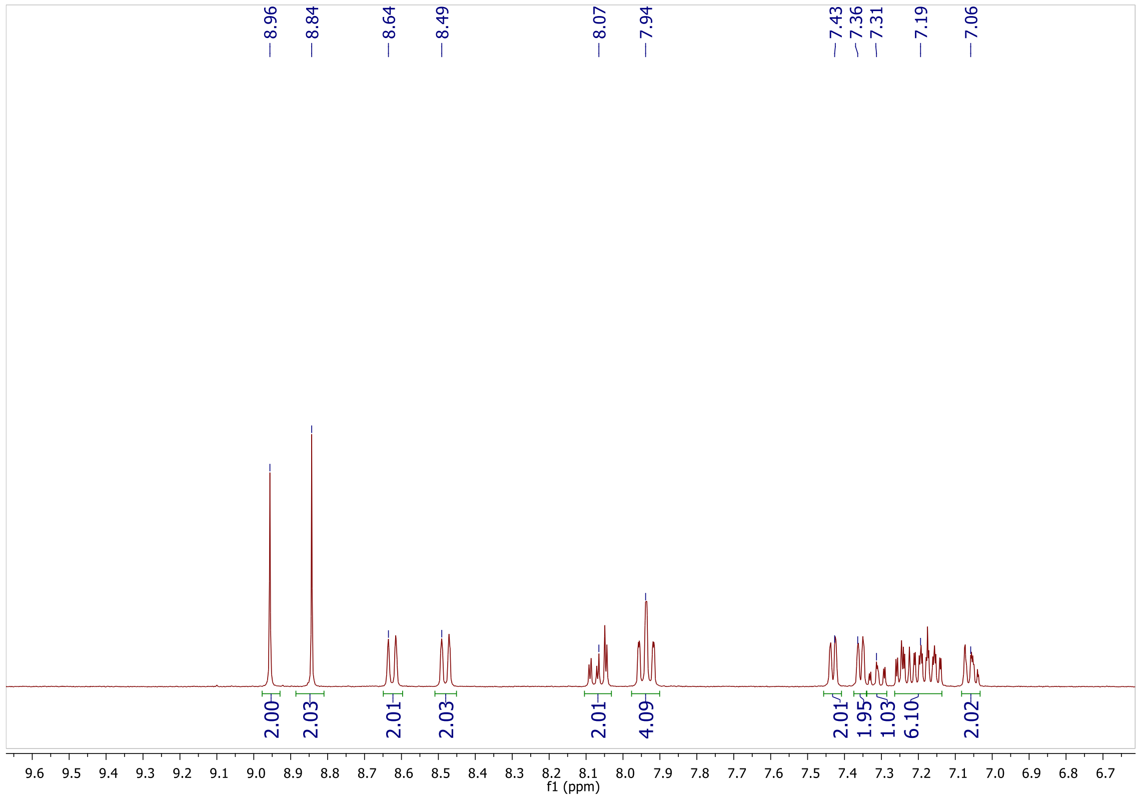

Figure S6 ^{1}H-NMR spectrum of **RuCl** in CD_3CN. (aromatic region)

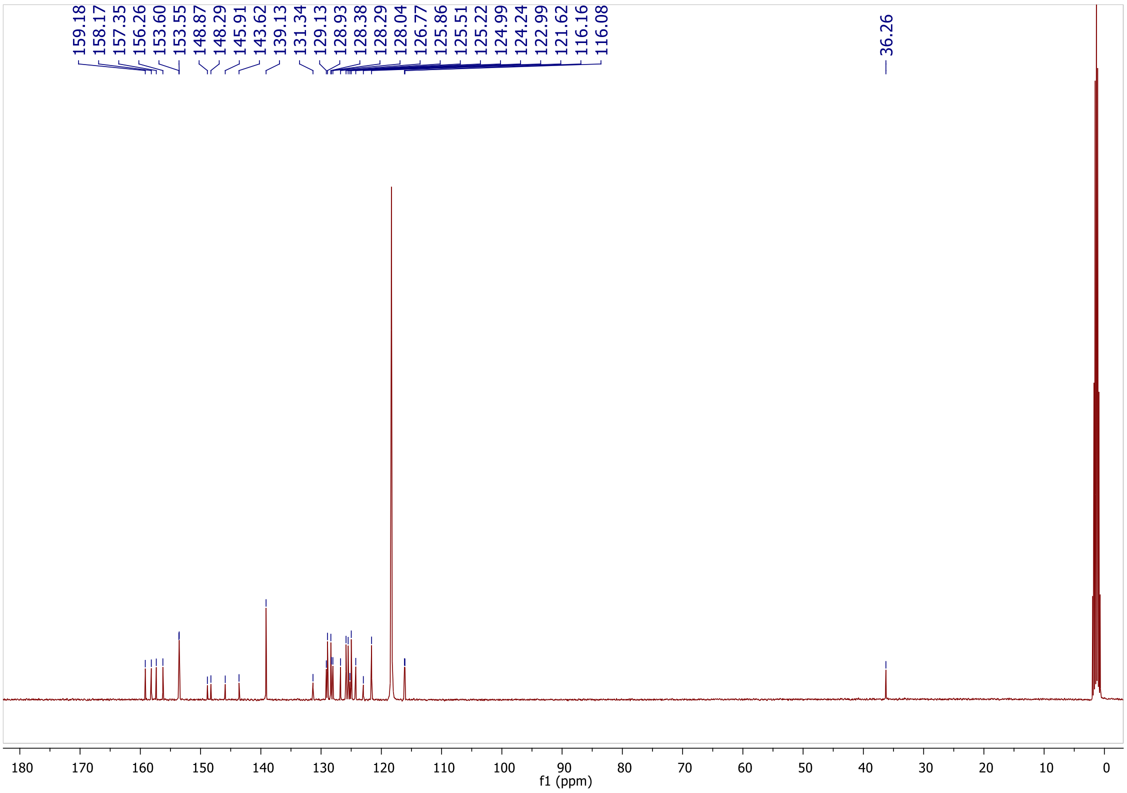

Figure S7 ^{13}C-NMR spectrum of **RuCl** in CD_3CN.

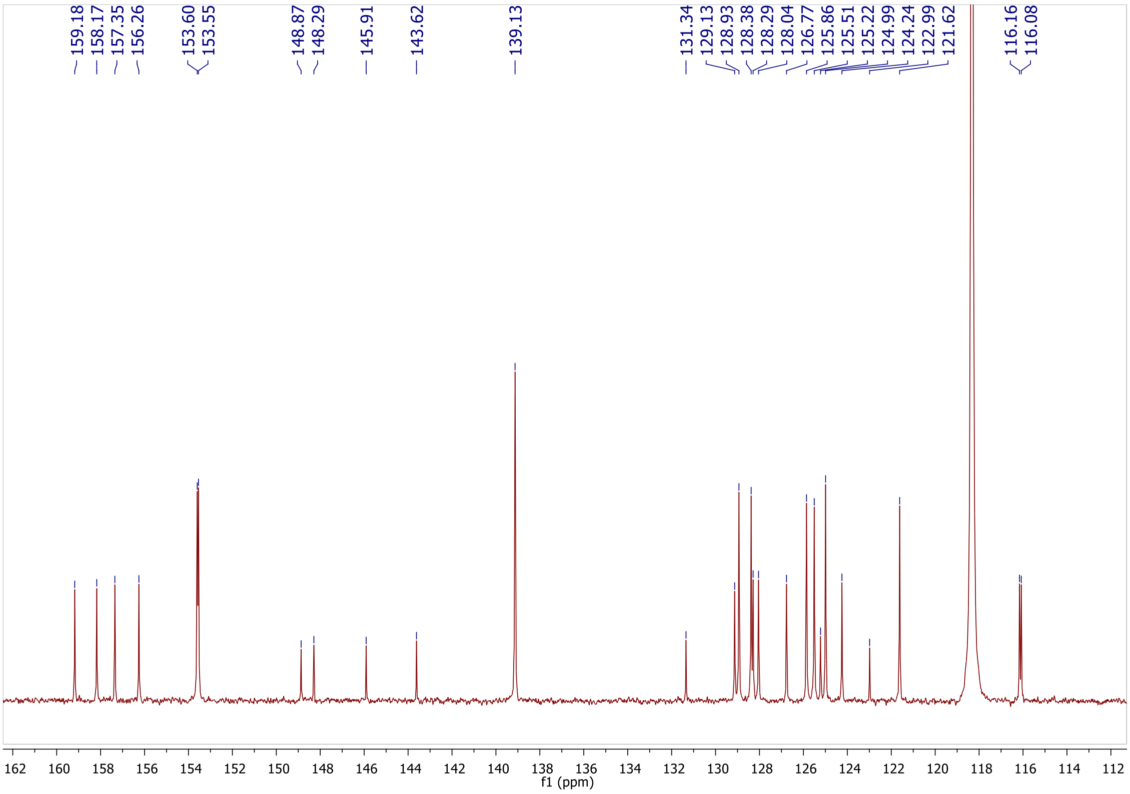

Figure S8 ^{13}C-NMR spectrum of **RuCl** in CD_3CN. (aromatic region)

HR-MS Spectrometry:

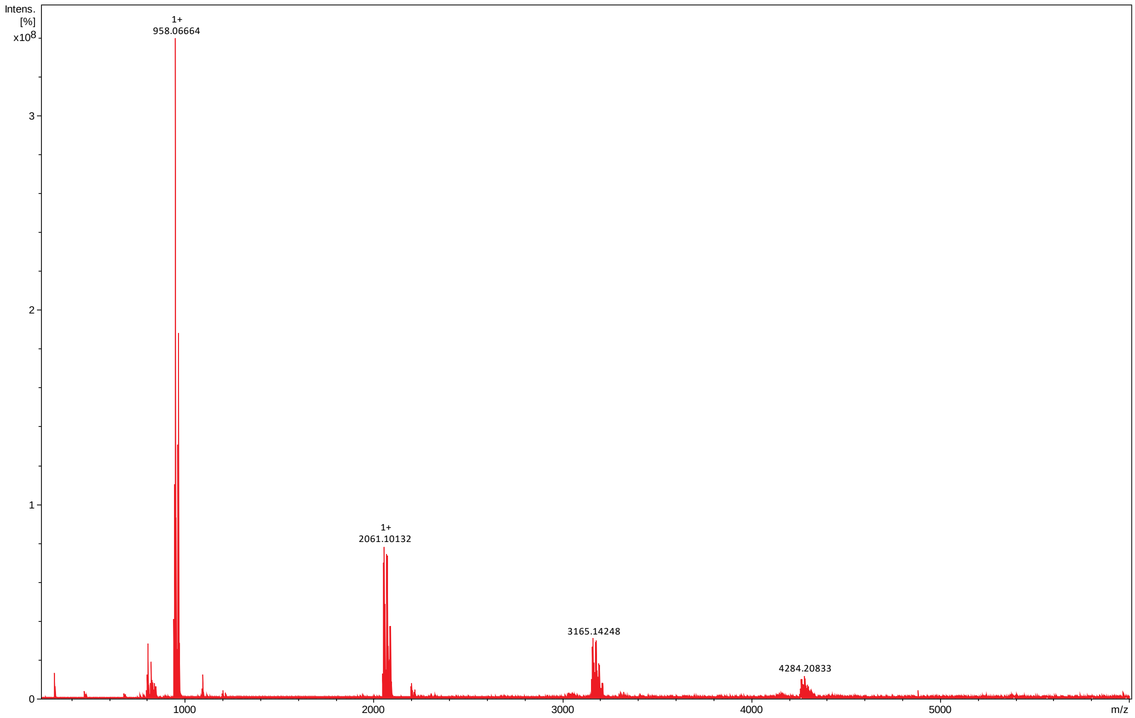

Figure S9 HR-MALDI-MS spectrum of **RuCl**.

High Resolution Mass Spectrometry (HRMS) was performed at the mass spectrometry service department of Ulm University by Dr. Markus Wunderlin, using a Fourier Transform Ion Cyclotron Resonance (FT-ICR) mass spectrometer solariX (Bruker Daltonics) equipped with a 7.0 T superconducting magnet and interfaced to an Apollo II Dual ESI/MALDI source. For all MALDI measurements trans-2-[3-(4-tert-butylphenyl)-2-methyl-2-propenylidene]malononitrile (DCTB) was used as the matrix. Spectra were analyzed with Compass Data Analysis Viewer Version 4.4.

NMR spectroscopy was performed on a Bruker Avance 400 MHz spectrometer at room temperature. The shift values are given in ppm and are referenced to the corresponding solvent residual peaks. MestReNova software Version v6.0.2-5475 was used to process the data. Coupling constants J are presented as absolute values in Hz. For the characterization of the NMR signals the following abbreviations are used: s = singlet, d = doublet, t = triplet, m = multiplet and dd = doublet of doublets, td = triplet of doublets.

Microwave reactions were performed in a MLS GmbH Mikrowellensystem Start 1500. The temperature was measured with an IR-TC infrared sensor.

Electrochemistry and excited state spectrometry:

PTZ-tpy-Ru-tpy-Cl (**RuCl**) was dissolved in degassed acetonitrile. The steady-state spectrum of the resulting solution in a cuvette with a pathlength of 1 cm showed an optical density of 0.3 in the blue edge of the Ruthenium-bis terpyridine **($[Ru(tpy)_2]^{2+}$)** metal-to-ligand charge-transfer (MLCT) band at 400nm[5], which was chosen for the central wavelength of the pump beam. A regenerative Ti:sapphire amplifier (Astrella, Coherent, USA) is used for the fundamental laser, delivering pulses of 5 mJ pulse energy at 1 kHz pulse repetition rate and 800 nm. The pulses have a duration of 80 fs. The pump wavelength is centered using a TOPAS-C Lightconversion, also from Coherent. A white light supercontinuum generated by focusing a fraction of the fundamental in a CaF_2 plate is used to probe the samples in a wide spectral range (340 to 750 nm). The pump beam is delayed in time with respect to the probe beam by means of an optical delay line and the polarization between probe and pump is set at the magic angle (54.7°). The power of the pump beam at the sample position was set to 0.35 to 0.40 mW. For all the experiments the stability of samples was ensured by recording the UV/Vis absorption spectra (JASCO V-670 spectrometer) at room temperature before and after the fs TA measurements.

The fs TA spectra were displayed after chirp correction. The fs TA data were processed and analyzed by a global multi-exponential parallel fit, using the Python Package KiMoPack.[6] A temporal window of 0.7 ps to 1.4 ps past time-zero was excluded in order to avoid contributions of the coherent-artifact region to the data analysis. Furthermore, a spectral band of 28 nm to 45 nm around the pump-wavelength is omitted from the data analysis due to pump-scatter in this spectral range.

The temperature settings were varied from 240 K up to 300 K in 5 K steps. For each temperature two experiments were performed. The mean value and the respective

derivation of the fitted kinetic rate constants as well as the measured sample temperature were used to determine the Arrhenius and Marcus parameters.

The CV in Figure S10 shows three distinct redox waves, including the Fc^+/Fc reference peak, which was shifted to around 0.25 V. The reversible peak in the positive potential area at 0.63 V was assigned to the monoelectronic oxidation of the donor unit PTZ. In case of inserting simulated ^{3}MLCT-^{3}ILCT as well as ^{3}MLCT-^{3}LLCT driving force (-0.20 and 0.03 eV, respectively) and redox center distance (6.6 and 11.9 Å, respectively) into Equation 4-5, two negative potentials -1.30 and -1.50 V are expected. Thus, the reversible peak in the negative potential area at -1.33 V was assigned to the monoelectronic reduction of both adjacent and remote terpyridyl ligands (Figure S10).

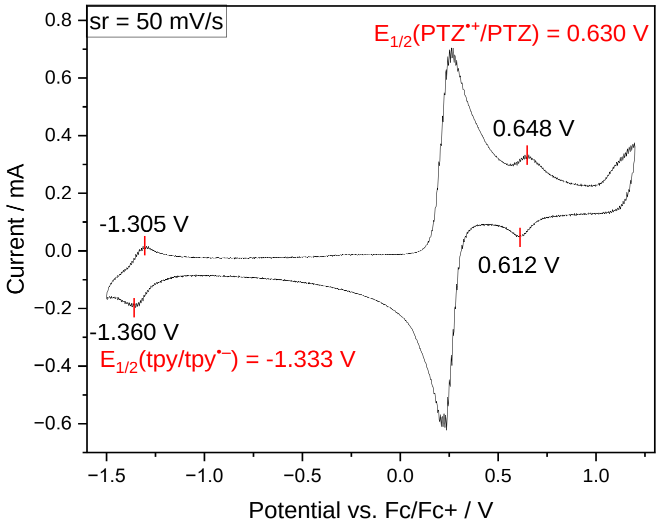

Figure S10. Second scan of cyclic voltammetry of the photodyad vs.Fc/Fc^+ reference electrode.

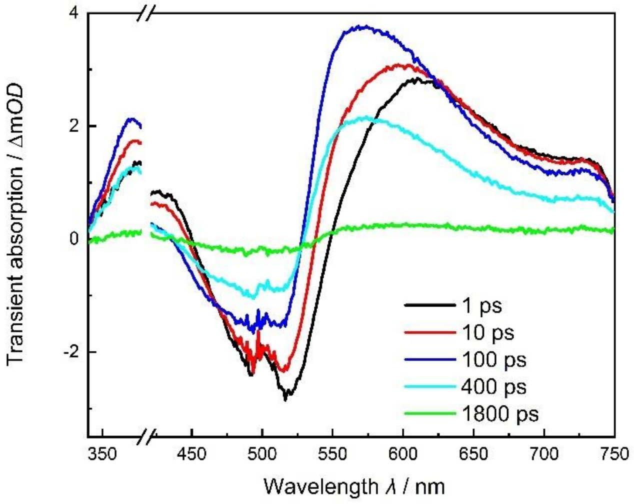

Figure S11. Femtosecond transient spectra at various delay times recorded at 240 K.

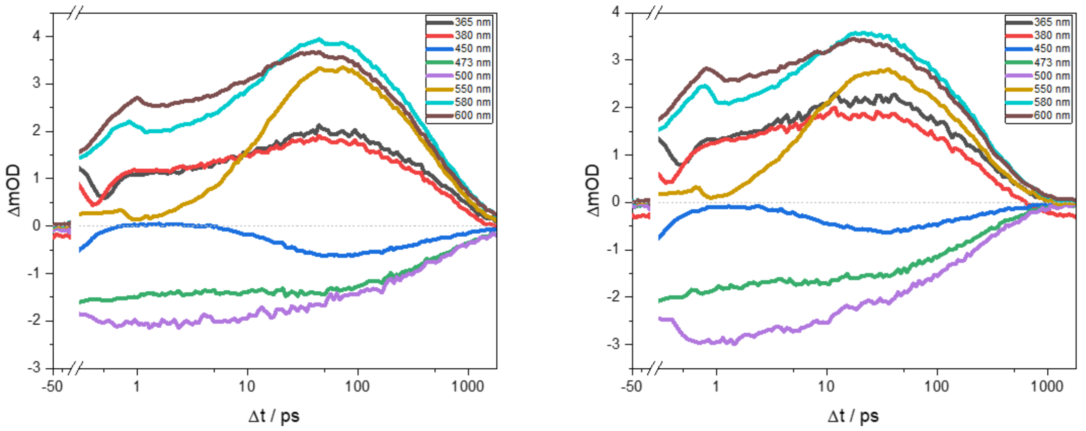

Figure S12. Kinetic traces of various wavelengths recorded at a) 240 K and b) 300 K, indicating a faster decay at higher temperature.

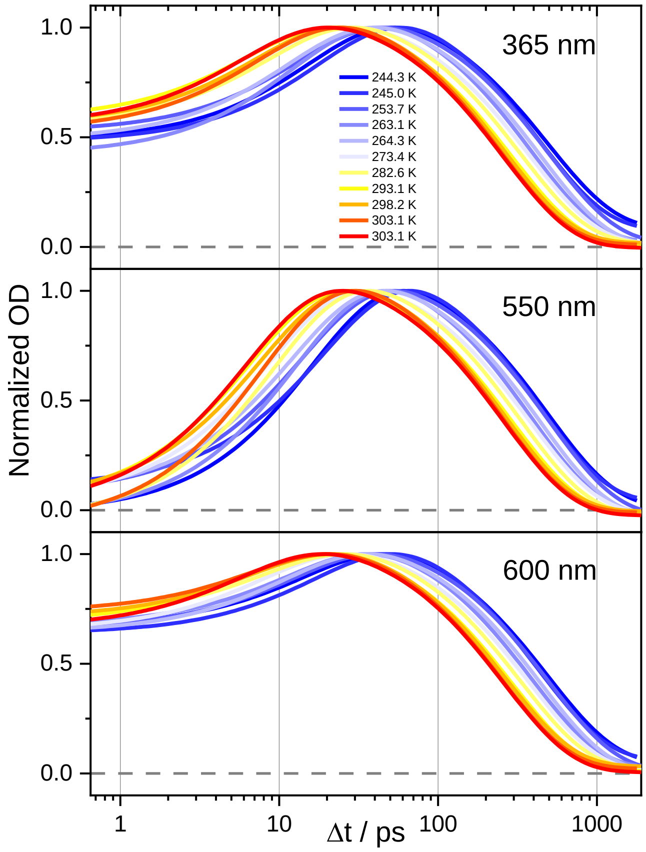

Figure S13. Kinetic traces of selected wavelengths recorded at different sample temperatures.

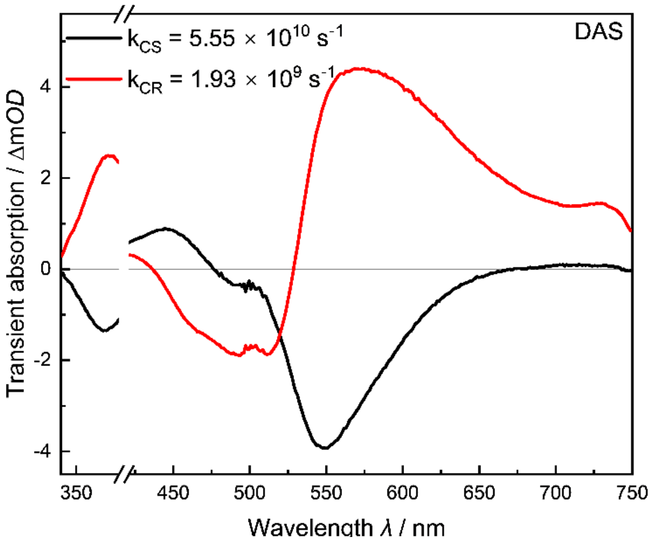

Figure S14. Decay associated spectra resulting from global fitting with two finite and one infinite component of the TA-Data recorded at 240 K.

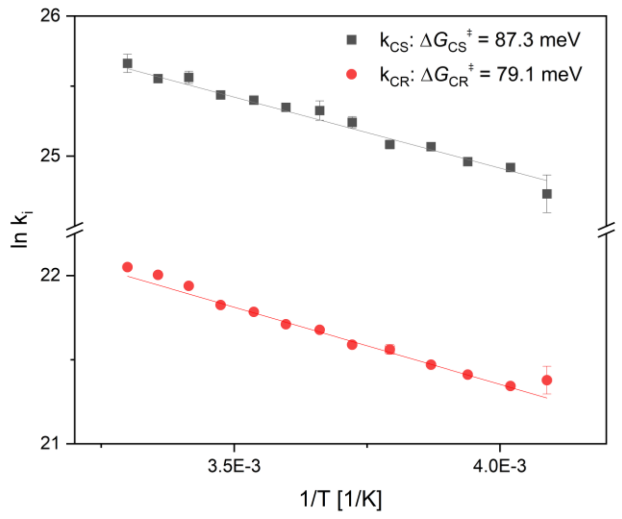

Figure S15. Arrhenius-Plot to receive the energy barrier from the slope of the linear regression. The charge-separation- (k_{CS}, black squares) and the charge-recombination process (k_{CR}, red dots).

Computational Section:

Table S1. A comparison of dihedral angles for **RuCl** in the singlet ground state (S_0), the ^{3}MLCT, ^{3}ILCT and ^{3}LLCT optimized geometries. Measured parameters are indicated in red.

Character	Dihedral Angle	S_0	^{3}MLCT	^{3}ILCT	^{3}LLCT
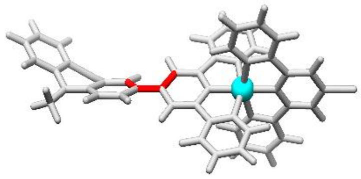	D	27.53	26.65	12.22	25.01

Table S2. A comparison of dihedral angles for **RuCl** in the singlet ground state (S_0), the ^{3}MLCT, ^{3}ILCT and ^{3}LLCT optimized geometries. Measured parameters are indicated in red.

Character	Angle	S_0	^{3}MLCT	^{3}ILCT	^{3}LLCT
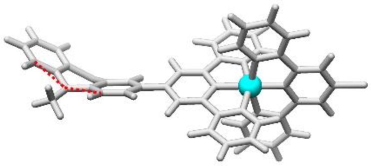	A	144.6	145.3	161.0	161.4

Table S3. A comparison of the Ru-N bond lengths for **RuCl** in the singlet ground state (S_0), the ^{3}MLCT, ^{3}ILCT and ^{3}LLCT optimized geometries. Measured parameters are indicated in red.

Character	Bond Length	S_0	^{3}MLCT	^{3}ILCT	^{3}LLCT
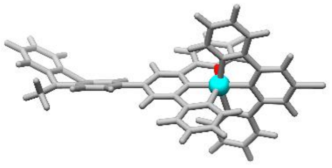	L_{1a}	2.08	2.08	2.09	2.07
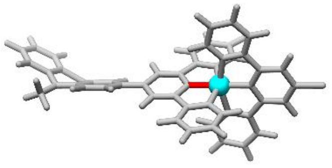	L_{1b}	1.99	2.04	1.97	1.96
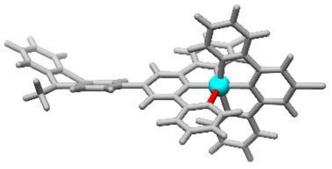	L_{1c}	2.08	2.08	2.09	2.07

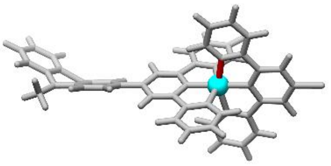	L_{2a}	2.08	2.09	2.08	2.09
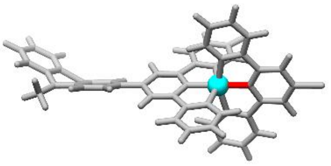	L_{2b}	1.99	1.93	1.99	2.01
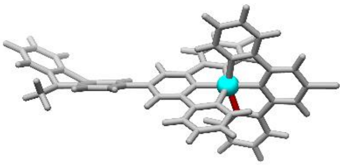	L_{2c}	2.08	2.09	2.08	2.07

Table S4. A comparison of angles between typ ligands and bite angles for **RuCl** in the singlet ground state (S_0), the ^{3}MLCT, ^{3}ILCT and ^{3}LLCT optimized geometries. Measured parameters are indicated in red.

Character	Angle	S_0	^{3}MLCT	^{3}ILCT	^{3}LLCT
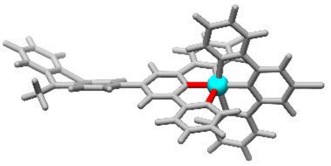	α_{1a}	79.0	77.3	79.0	79.7
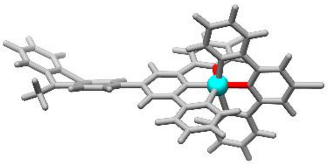	α_{1b}	101.1	102.6	101.3	100.3
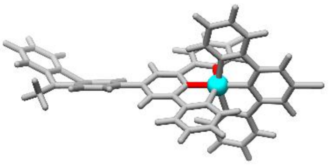	α_{1c}	79.00	77.3	79.1	79.7

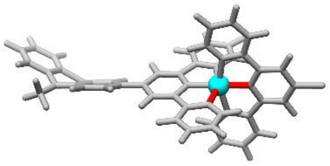	α_{1d}	100.9	102.8	100.7	100.3
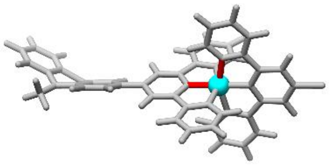	α_{2a}	100.8	99.7	100.3	102.1
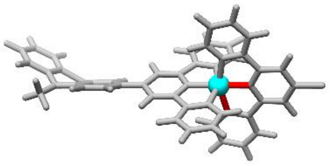	α_{2b}	79.2	80.2	79.0	79.6
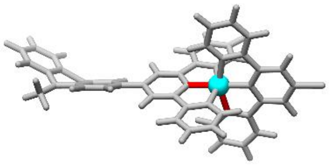	α_{2c}	100.9	100.0	101.6	100.5
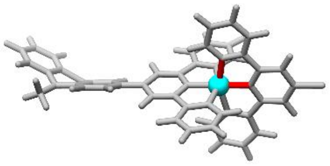	α_{2d}	79.2	80.2	79.1	77.9
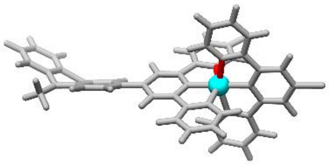	α_{3a}	92.2	92.4	92.3	92.3
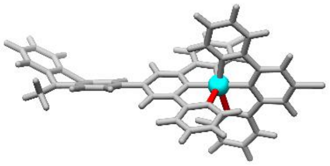	α_{3b}	92.2	92.3	92.5	91.5

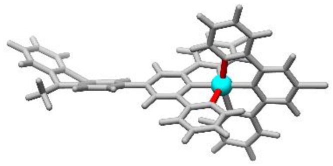	α$_{3c}$	91.9	91.9	91.8	92.7
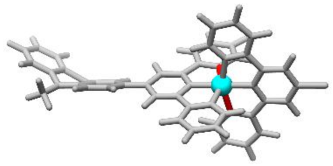	α$_{3d}$	91.9	92.0	91.8	91.6

Table S5. A comparison of dihedral angles for **RuCl** in the singlet ground state (S_0), the ^{3}MLCT, ^{3}ILCT and ^{3}LLCT optimized geometries. Measured parameters are indicated in red.

Character	Dihedral Angle	S_0	^{3}MLCT	^{3}ILCT	^{3}LLCT
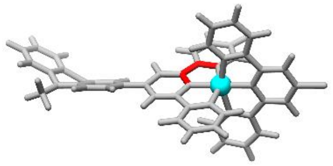	δ$_{1a}$	0.14	0.15	-0.89	-0.30
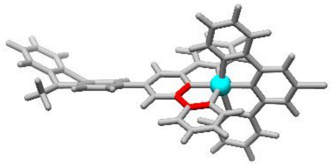	δ$_{1b}$	0.14	0.18	1.40	0.57
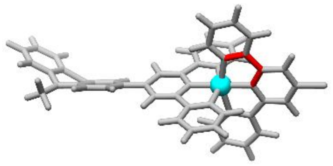	δ$_{2a}$	0.07	0.05	-0.07	0.10
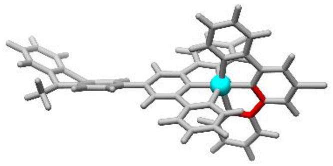	δ$_{2b}$	-0.11	-0.10	-0.21	0.09

Table S6. Simulated excited state properties of the low-lying bright singlet excited states of **RuCl** in CH_3CN such as excitation energies (in eV), excitation wave lengths (in nm), oscillator strengths, spin contamination, MO pairs, leading transitions as represented by charge density differences (CDDs; charge transfer takes place from red to blue). All results were obtained using the B3LYP functional as implemented in Gaussian 16.

Transition ($S_0 \rightarrow S_x$)	ΔE / eV	λ / nm	f	$\langle s^2 \rangle$	Character
S_2	2.26	549	0.3007	0.000	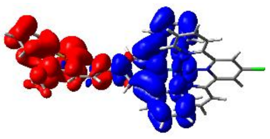
S_{10}	2.77	448	0.0445	0.000	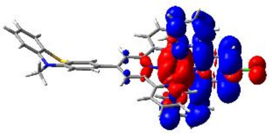
S_{11}	2.77	448	0.0708	0.000	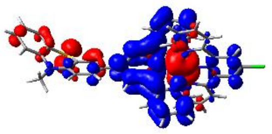
S_{13}	2.84	436	0.2308	0.000	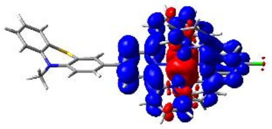
S_{15}	2.93	424	0.0341	0.000	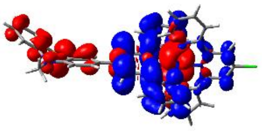
S_{17}	3.26	380	0.1568	0.000	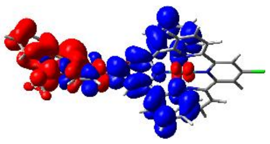
S_{19}	3.40	365	0.0670	0.000	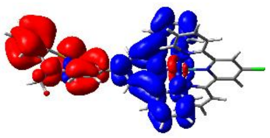

Table S7. Simulated excited state properties of the bright triplet excited states of **RuCl** within the ^{3}MLCT equilibrium structure in CH_3CN such as excitation energies (in eV), excitation wave lengths (in nm), oscillator strengths, spin contamination, MO pairs, leading transitions as represented by charge density differences (CDDs; charge transfer takes place from red to blue). All results were obtained using the B3LYP functional as implemented in Gaussian 16.

Transition ($T_1 \rightarrow T_x$)	ΔE / eV	λ / nm	f	$\langle s^2 \rangle$	Character
T_{10}	1.39	893	0.0426	2.098	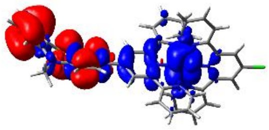
T_{27}	2.43	510	0.2024	2.300	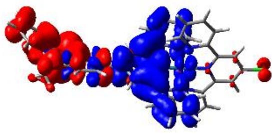
T_{53}	3.21	386	0.0718	2.541	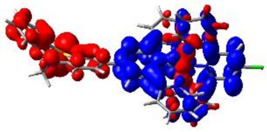
T_{55}	3.26	380	0.1574	2.237	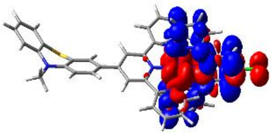
T_{57}	3.29	377	0.0596	2.435	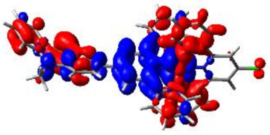

Table S8. Simulated excited state properties of the bright triplet excited states of **RuCl** within the ³ILCT equilibrium structure in CH_3CN such as excitation energies (in eV), excitation wave lengths (in nm), oscillator strengths, spin contamination, MO pairs, leading transitions as represented by charge density differences (CDDs; charge transfer takes place from red to blue). All results were obtained using the B3LYP functional as implemented in Gaussian 16.

Transition ($T_1 \rightarrow T_x$)	ΔE / eV	λ / nm	f	$\langle s^2 \rangle$	Character
T_8	1.21	1025	0.6919	2.028	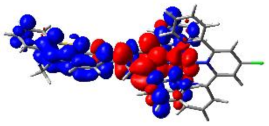
T_{11}	1.61	770	0.0773	2.042	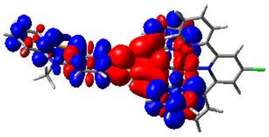
T_{26}	2.39	519	0.1701	2.046	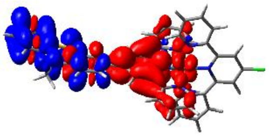
T_{39}	2.81	441	0.1291	2.054	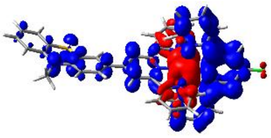

Table S9. Simulated excited state properties of the bright triplet excited states of **RuCl** within the ^{3}LLCT equilibrium structure in CH_3CN such as excitation energies (in eV), excitation wave lengths (in nm), oscillator strengths, spin contamination, MO pairs, leading transitions as represented by charge density differences (CDDs; charge transfer takes place from red to blue). All results were obtained using the B3LYP functional as implemented in Gaussian 16.

Transition ($T_1 \rightarrow T_x$)	ΔE / eV	λ / nm	f	$\langle s^2 \rangle$	Character
T_{13}	1.61	769	0.0178	2.060	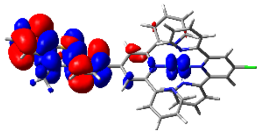
T_{16}	1.84	675	0.0306	3.742	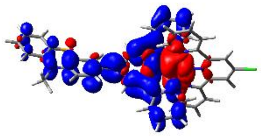
T_{21}	2.17	572	0.0509	2.055	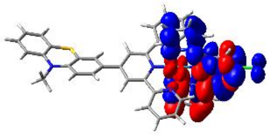
T_{28}	2.40	517	0.1881	2.168	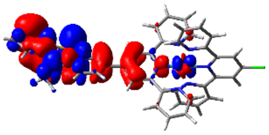
T_{29}	2.45	506	0.0768	2.090	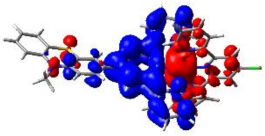
T_{33}	2.58	481	0.0783	2.082	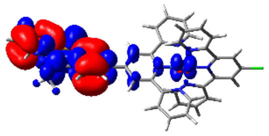
T_{36}	2.66	466	0.1640	2.515	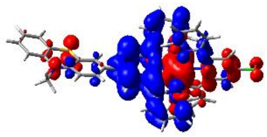
T_{38}	2.72	457	0.0841	2.566	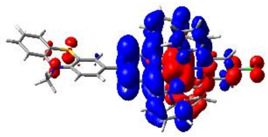

Transition ($T_1 \rightarrow T_x$)	ΔE / eV	λ / nm	f	⟨s²⟩	Character
T_{40}	2.76	449	0.0522	2.226	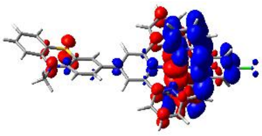
T_{52}	3.11	399	0.0524	2.289	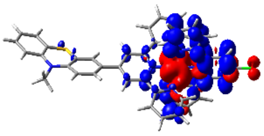
T_{55}	3.18	390	0.0674	3.226	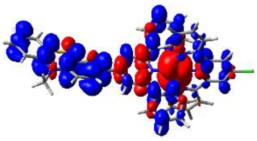
T_{56}	3.21	386	0.0425	2.412	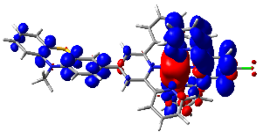
T_{63}	3.36	369	0.0488	2.265	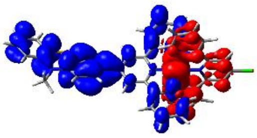
T_{66}	3.43	361	0.0304	2.510	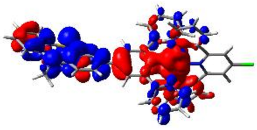

Table S10. The electronic couplings (V_{DA} in eV) obtained by the generalized Mulliken–Hush (GMH) method as well as the fragment charge difference (FCD) approach for **RuCl** complex.

Method	^{3}MLCT-^{3}ILCT coupling along ^{3}MLCT-^{3}ILCT coordinate	^{3}MLCT-^{3}LLCT coupling along ^{3}MLCT-^{3}LLCT coordinate
GMH	1.50×10^{-2}	4.50×10^{-4}
FCD	1.50×10^{-2}	4.50×10^{-4}

Table S11. Spin-orbit coupling (SOCs in cm^{-1}) matrix elements at the Franck-Condon point between optically accessible singlet excited states and spin forbidden triplet states of **RuCl**. Energies (in eV) and oscillator strengths of the respective singlet spin-free transitions (from S_0) are indicated in parenthesis.

$\langle T_j \vert H_{SOC} \vert S_i \rangle$ / cm^{-1}	T_1 (1.95) ^{3}ILCT	T_2 (2.06) ^{3}MLCT	T_3 (2.19) ^{3}ILCT/^{3}MLCT	T_4 (2.21) ^{3}MLCT	T_5 (2.28) ^{3}MLCT	T_6 (2.34) ^{3}ILCT/^{3}MLCT	T_7 (2.37) ^{3}ILCT	T_8 (2.43) ^{3}MLCT	T_9 (2.44) ^{3}MLCT	T_{10} (2.44) ^{3}ILCT/^{3}MLCT	T_{11} (2.53) ^{3}MLCT	T_{12} (2.56) ^{3}MLCT	T_{13} (2.58) ^{3}MLCT/^{3}ILCT	T_{14} (2.76) ^{3}MLCT	T_{15} (2.79) ^{3}MLCT	T_{16} (2.83) ^{3}MLCT	T_{17} (2.92) ^{3}ILCT	T_{18} (3.11) ^{3}MLCT	T_{19} (3.17) ^{3}MLCT/^{3}ILCT	T_{20} (3.21) ^{3}MC
S_2, ^{1}ILCT (2.32;0.363)	3	5	100	47	141	4	2	122	6	25	12	4	2	11	14	22	1	1	1	25
S_{10}, ^{1}MLCT (2.70;0.047)	0	47	34	4	32	85	74	23	258	180	4	90	16	180	155	2	9	132	21	30
S_{11}, ^{1}MLCT (2.72;0.056)	2	2	99	8	290	14	8	238	17	82	126	7	27	12	158	118	11	2	4	124
S_{12}, ^{1}MLCT (2.78;0.172)	1	1	125	165	53	143	16	6	23	18	141	11	80	234	44	265	10	11	5	69
S_{15}, ^{1}MLCT (2.90;0.034)	24	19	27	2	2	2	7	32	126	27	70	209	25	6	8	267	42	24	123	31
S_{17}, ^{1}ILCT (3.29;0.173)	1	0	1	18	14	5	1	15	3	10	12	1	8	1	5	8	0	1	2	8
S_{19}, ^{1}ILCT (3.46;0.076)	6	2	3	5	60	15	5	53	5	12	4	2	6	5	6	11	0	1	3	14

Table S12. Spin-orbit coupling (SOCs in cm^{-1}) matrix elements at the equilibrated geometry of ^{3}MLCT state between optically accessible singlet excited states and spin forbidden triplet states of **RuCl**. Energies (in eV) and oscillator strengths of the respective singlet spin-free transitions (from S_0) are indicated in parenthesis; all energies are shown relative to the singlet ground state within the ^{3}MLCT geometry.

$\langle T_j \| H_{SOC} \| S_i \rangle$ / cm^{-1}	T_1 (1.65) ^{3}MLCT	T_2 (2.01) ^{3}ILCT	T_3 (2.08) ^{3}MLCT	T_4 (2.12) ^{3}ILCT/^{3}MLCT	T_5 (2.33) ^{3}MLCT	T_6 (2.33) ^{3}MLCT	T_7 (2.34) ^{3}MLCT	T_8 (2.38) ^{3}LLCT	T_9 (2.42) ^{3}MLCT	T_{10} (2.46) ^{3}MLCT	T_{11} (2.47) ^{3}ILCT/^{3}MLCT	T_{12} (2.48) ^{3}ILCT/^{3}MLCT	T_{13} (2.61) ^{3}MLCT	T_{14} (2.71) ^{3}MLCT	T_{15} (2.84) ^{3}MLCT	T_{16} (2.88) ^{3}MC	T_{17} (2.92) ^{3}ILCT	T_{18} (3.11) ^{3}MC	T_{19} (3.12) ^{3}MLCT	T_{20} (3.16) ^{3}MC
S_3, ^{1}ILCT (2.30;0.402)	14	4	5	122	98	9	110	15	4	1	10	9	3	3	17	8	1	8	2	66
S_6, ^{1}MLCT (2.50;0.036)	2	9	311	218	76	154	11	18	22	5	90	144	10	56	139	35	13	109	7	206
S_{10}, ^{1}MLCT (2.56;0.074)	54	11	4	43	21	6	26	71	127	224	128	25	67	2	253	9	7	43	133	139
S_{12}, ^{1}MLCT (2.77;0.149)	1	2	8	104	191	217	228	99	13	5	89	33	5	6	218	49	15	111	5	52
S_{13}, ^{1}MLCT (2.82;0.040)	0	2	85	9	127	115	194	70	1	8	172	34	10	255	137	214	69	84	5	55
S_{15}, ^{1}MLCT (3.01;0.031)	11	20	38	30	21	40	3	3	10	101	2	28	233	276	2	23	48	25	17	5
S_{17}, ^{1}ILCT (3.29;0.147)	0	1	19	3	15	11	15	5	1	2	10	4	2	5	6	2	1	4	1	7
S_{19}, ^{1}ILCT (3.43;0.075)	3	4	8	13	61	7	69	23	6	4	16	7	2	16	2	13	4	7	1	4

Table S13. Spin-orbit coupling (SOCs in cm^{-1}) matrix elements at the equilibrated geometry of ^{3}ILCT state between optically accessible singlet excited states and spin forbidden triplet states of **RuCl**. Energies (in eV) and oscillator strengths of the respective singlet spin-free transitions (from S_0) are indicated in parenthesis; all energies are shown relative to the singlet ground state within the ^{3}ILCT geometry.

$\langle T_j \vert H_{SOC} \vert S_i \rangle$ / cm^{-1}	T_1 (1.50) ^{3}ILCT	T_2 (1.94) ^{3}LLCT	T_3 (1.95) ^{3}ILCT/^{3}LLCT	T_4 (2.00) ^{3}MLCT	T_5 (2.10) ^{3}MLCT/^{3}ILCT	T_6 (2.12) ^{3}LLCT	T_7 (2.20) ^{3}MLCT	T_8 (2.21) ^{3}LLCT	T_9 (2.32) ^{3}LLCT	T_{10} (2.43) ^{3}MLCT	T_{11} (2.44) ^{3}LLCT/^{3}MLCT	T_{12} (2.45) ^{3}ILCT/^{3}MLCT	T_{13} (2.53) ^{3}MLCT	T_{14} (2.57) ^{3}MLCT	T_{15} (2.66) ^{3}MLCT/^{3}ILCT	T_{16} (2.69) ^{3}MLCT/^{3}ILCT	T_{17} (2.83) ^{3}MLCT	T_{18} (2.97) ^{3}IL	T_{19} (3.01) ^{3}ILCT	T_{20} (3.06) ^{3}ILCT
S_1, ^{1}ILCT (1.91;0.413)	3	68	23	14	10	61	91	26	95	3	16	7	11	4	7	12	15	3	2	7
S_9, ^{1}MLCT (2.57;0.117)	4	142	59	5	9	46	255	188	278	8	97	23	76	2	102	4	72	6	4	5
S_{12}, ^{1}MLCT (2.71;0.046)	3	28	53	28	41	133	6	21	31	228	52	139	16	83	145	217	18	8	11	3
S_{13}, ^{1}MLCT (2.77;0.330)	2	78	84	8	9	29	16	131	51	25	86	24	128	2	181	44	186	6	9	8
S_{15}, ^{1}MLCT/^{1}ILCT (2.83;0.052)	20	24	11	7	37	7	9	44	38	135	30	20	65	197	34	5	264	41	11	22

Table S14. Spin-orbit coupling (SOCs in cm^{-1}) matrix elements at the equilibrated geometry of ^{3}LLCT state between optically accessible singlet excited states and spin forbidden triplet states of **RuCl**. Energies (in eV) and oscillator strengths of the respective singlet spin-free transitions (from S_0) are indicated in parenthesis; all energies are shown relative to the singlet ground state within the ^{3}LLCT geometry.

$\langle T_j \vert H_{SOC} \vert S_i \rangle$ / cm^{-1}	T_1 (1.68) ^{3}ILCT	T_2 (1.70) ^{3}LLCT	T_3 (1.91) ^{3}MLCT	T_4 (1.98) ^{3}MLCT	T_5 (1.99) ^{3}ILCT/^{3}LLCT	T_6 (2.09) ^{3}ILCT/^{3}LLCT	T_7 (2.17) ^{3}MLCT	T_8 (2.18) ^{3}MLCT/^{3}ILCT	T_9 (2.28) ^{3}MLCT	T_{10} (2.39) ^{3}MLCT	T_{11} (2.45) ^{3}ILCT	T_{12} (2.49) ^{3}MLCT	T_{13} (2.54) ^{3}MLCT	T_{14} (2.47) ^{3}MLCT	T_{15} (2.63) ^{3}MLCT	T_{16} (2.66) ^{3}MLCT	T_{17} (2.89) ^{3}MLCT	T_{18} (2.90) ^{3}MLCT	T_{19} (2.94) ^{3}IL	T_{20} (2.99) ^{3}ILCT
S_2, ^{1}ILCT (1.96;0.286)	2	55	13	21	25	43	28	7	76	6	5	79	9	5	4	9	12	4	5	3
S_8, ^{1}MLCT (2.48;0.097)	13	137	158	247	56	92	250	35	44	6	2	88	136	24	131	38	14	114	2	3
S_{10}, ^{1}MLCT (2.66;0.121)	5	27	17	16	11	102	96	15	247	76	11	196	211	50	203	120	59	7	7	7
S_{13}, ^{1}MLCT (2.80;0.199)	1	29	7	82	82	88	38	26	24	197	23	43	85	46	215	149	211	10	21	7
S_{14}, ^{1}MLCT (2.82;0.040)	21	11	5	30	11	14	29	41	9	87	55	33	57	226	11	19	259	96	34	3
S_{15}, ^{1}ILCT (2.84;0.041)	3	9	7	31	29	19	12	12	23	46	21	10	24	58	51	76	109	32	10	4

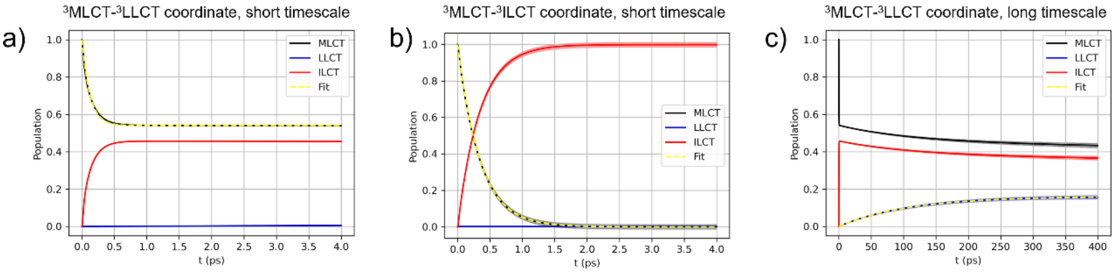

Figure S16. Population densities of ^{3}MLCT (black), ^{3}LLCT (blue) and ^{3}ILCT (red) states according to dissipative quantum dynamics starting from the "hot ^{3}MLCT" state; yellow dashed lines depict the fits used to determine reaction rate constants. Fast population transfer within initial 4 ps, a) along the ^{3}MLCT-^{3}LLCT coordinate, and b) along the ^{3}MLCT-^{3}ILCT coordinate. c) Slow population transfer within 400 ps along the ^{3}MLCT-^{3}LLCT coordinate.

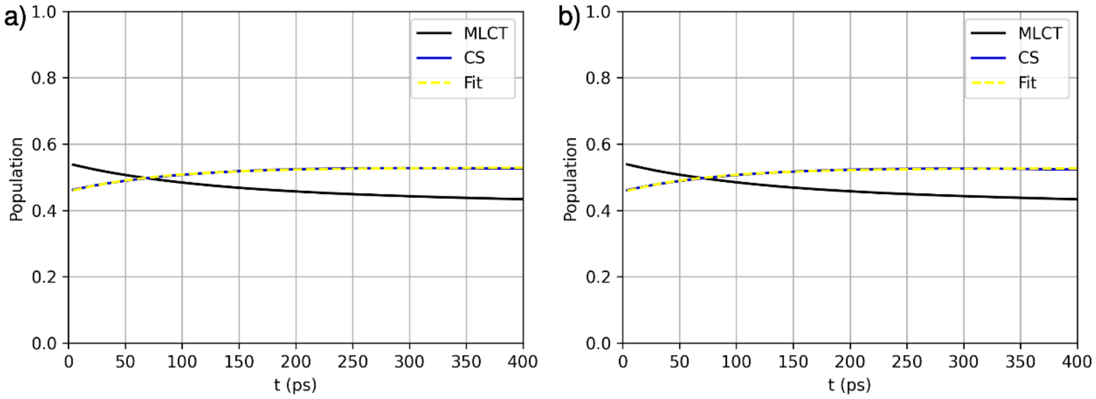

Figure S17. Population densities of the ^{3}MLCT (black), and the ^{3}CS (blue, ^{3}LLCT + ^{3}ILCT) states according to dissipative quantum dynamics starting from the equilibrated ^{3}MLCT state (a) and the "hot ^{3}MLCT" state (b); yellow dashed lines depict the fit used to determine reaction rate constants. Population transfer is shown within 4 ps to 400 ps along the ^{3}MLCT-^{3}LLCT coordinate.

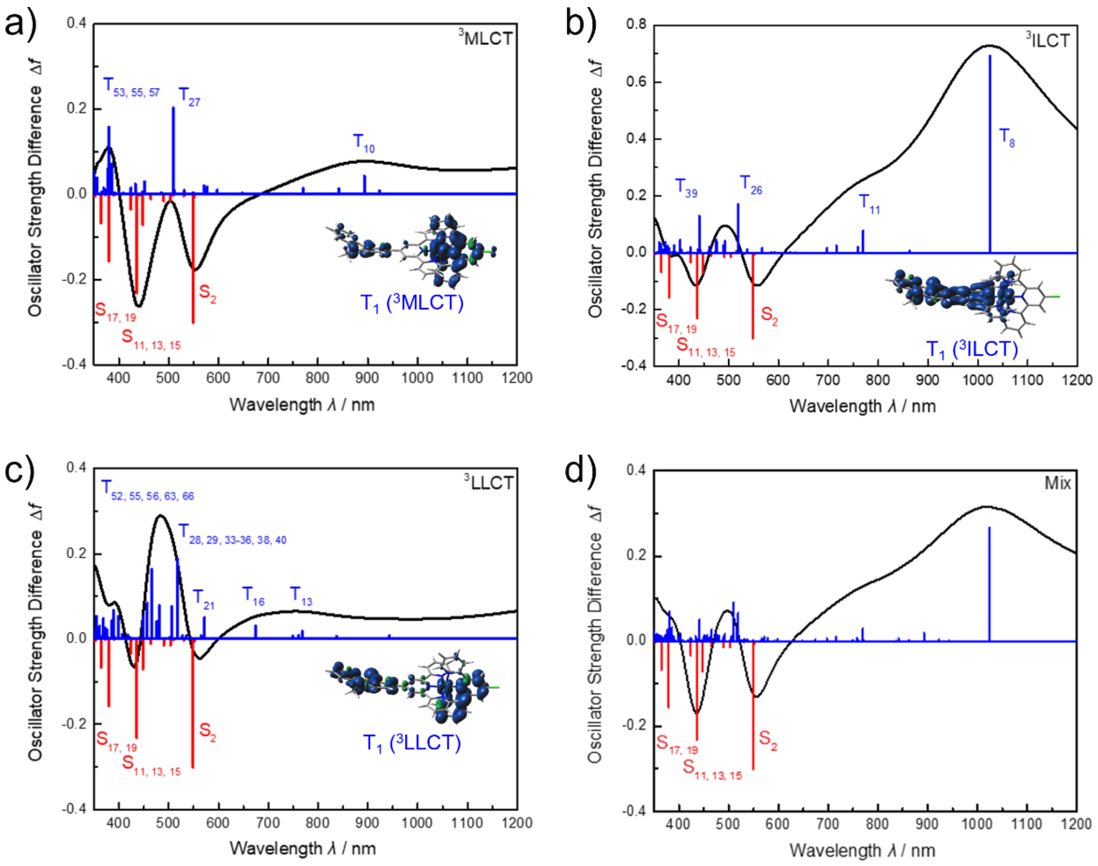

Figure S18. Simulated transient absorption spectra within a) ^{3}MLCT, b) ^{3}ILCT, c) ^{3}LLCT equilibrium geometries, respectively, and obtained at the TDDFT level of theory. Spin densities are shown to visualize the nature of the respective triplet ground state species d) Simulated spectrum including excited-state absorption contributions from ^{3}MLCT (43%), ^{3}ILCT (37%), and ^{3}LLCT (16%) states. In contrast to Figure 6 within the main text, the spectral range was extended to 1200 nm.

References

[1] a) M. Kolek, F. Otteny, P. Schmidt, C. Mück-Lichtenfeld, C. Einholz, J. Becking, E. Schleicher, M. Winter, P. Bieker, B. Esser, *Energy Environ. Sci.* **2017**, *10*, 2334-2341; b) K. Barthelmes, A. Winter, U. S. Schubert, *Eur. J. Inorg. Chem.* **2016**, *2016*, 5132-5142.

[2] B. P. Sullivan, J. M. Calvert, T. J. Meyer, *Inorg. Chem.* **1980**, *19*, 1404-1407.

[3] B. Schulze, D. Escudero, C. Friebe, R. Siebert, H. Görls, S. Sinn, M. Thomas, S. Mai, J. Popp, B. Dietzek, L. González, U. S. Schubert, *Chem. Eur. J.* **2012**, *18*, 4010-4025.

[4] Y. Luo, J. H. Tran, M. Wächtler, M. Schulz, K. Barthelmes, A. Winter, S. Rau, U. S. Schubert, B. Dietzek, *ChemComm.* **2019**, *55*, 2273-2276.

[5] J. T. Hewitt, P. J. Vallett, N. H. Damrauer, *J. Phys. Chem. A.* **2012**, *116*, 11536-11547.

[6] C. Müller, T. Pascher, A. Eriksson, P. Chabera, J. Uhlig, *J. Phys. Chem. A.* **2022**, *126*, 4087-4099.
